# Data-Driven Ensemble Docking to Map Molecular Interactions of Steroid Analogs with Hepatic Organic Anion Transporting Polypeptides

**DOI:** 10.1021/acs.jcim.1c00362

**Published:** 2021-06-09

**Authors:** Alzbeta Tuerkova, Orsolya Ungvári, Réka Laczkó-Rigó, Erzsébet Mernyák, Gergely Szakács, Csilla Özvegy-Laczka, Barbara Zdrazil

**Affiliations:** # University of Vienna, Department of Pharmaceutical Sciences, Division of Pharmaceutical Chemistry, Althanstraße 14, A-1090 Vienna, Austria; ‡ Drug Resistance Research Group, Institute of Enzymology, RCNS, Eötvös Loránd Research Network, H-1117, Budapest, Magyar tudósok krt. 2, Hungary; § Department of Organic Chemistry, University of Szeged, Dóm tér 8, H-6720 Szeged, Hungary; ∥ Department of Medicine I, Institute of Cancer Research, Comprehensive Cancer Center, Medical University of Vienna, A-1090 Vienna, Austria

## Abstract

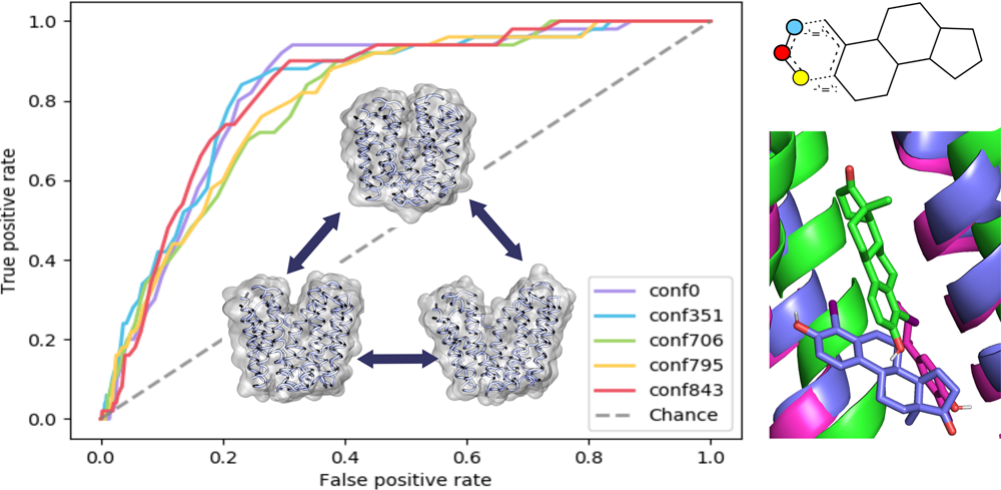

Hepatic organic anion transporting polypeptides—OATP1B1, OATP1B3, and OATP2B1—are expressed at the basolateral membrane of hepatocytes, being responsible for the uptake of a wide range of natural substrates and structurally unrelated pharmaceuticals. Impaired function of hepatic OATPs has been linked to clinically relevant drug–drug interactions leading to altered pharmacokinetics of administered drugs. Therefore, understanding the commonalities and differences across the three transporters represents useful knowledge to guide the drug discovery process at an early stage. Unfortunately, such efforts remain challenging because of the lack of experimentally resolved protein structures for any member of the OATP family. In this study, we established a rigorous computational protocol to generate and validate structural models for hepatic OATPs. The multistep procedure is based on the systematic exploration of available protein structures with shared protein folding using normal-mode analysis, the calculation of multiple template backbones from elastic network models, the utilization of multiple template conformations to generate OATP structural models with various degrees of conformational flexibility, and the prioritization of models on the basis of enrichment docking. We employed the resulting OATP models of OATP1B1, OATP1B3, and OATP2B1 to elucidate binding modes of steroid analogs in the three transporters. Steroid conjugates have been recognized as endogenous substrates of these transporters. Thus, investigating this data set delivers insights into mechanisms of substrate recognition. In silico predictions were complemented with in vitro studies measuring the bioactivity of a compound set on OATP expressing cell lines. Important structural determinants conferring shared and distinct binding patterns of steroid analogs in the three transporters have been identified. Overall, this comparative study provides novel insights into hepatic OATP-ligand interactions and selectivity. Furthermore, the integrative computational workflow for structure-based modeling can be leveraged for other pharmaceutical targets of interest.

## Introduction

Solute carriers (SLC) are increasingly recognized for their pivotal role in compound pharmacokinetics, given their involvement in drug absorption, disposition, metabolism, elimination, clinically relevant drug–drug interactions, and related organ toxicities.^1,2^ Here, we focus on a triad of organic anion transporting polypeptides of the SLCO (SLC21) superfamily. OATP1B1 (*SLCO1B1* gene), OATP1B3 (*SLCO1B3* gene), and OATP2B1 (*SLCO2B1* gene) are expressed at the basolateral membrane of hepatocytes, mediating the cellular uptake of a broad spectrum of endogenous substrates and xenobiotics.^3−5^ Endogenous compounds include bilirubin, bile acids, steroid conjugates, and hormones. Drugs transported by hepatic OATPs are structurally and functionally quite heterogeneous, such as statins (pitavastatin, rosuvastatin, fluvastatin),^6^ antihistamines (fexofenadine),^7^ anticancer agents (SN-38, paclitaxel, imatinib),^8^ antibiotics (rifampicin, clarithromycin, benzylpenicillin),^9^ or anti-inflammatory drugs (ibuprofen, diclofenac, lumiracoxib).^10^ OATP-mediated drug–drug interactions represent a challenge for drug development. Therefore, the U.S. Food and Drug Administration recommends testing of novel drug candidates for their potential interactions with hepatic OATPs. The computational prediction of whether a certain drug might interact with hepatic OATPs is a promising approach at the early stage in the drug discovery pipeline to minimize the risk of attrition. However, these efforts remain challenging, mainly because of the lack of experimental protein structures for any member of the OATP family. In general, the sequence identity of hepatic OATPs to the closest structural analogs that have been resolved experimentally, does not exceed 16%, which makes the generation of the high-quality structural models a nontrivial task. Homology modeling becomes error-prone below 30% sequence identity (referred to as “twilight zone” protein modeling). In recognition of this challenging modeling task, we decided to use a threading approach by applying secondary structure prediction to identify the closest structural analog to be used as a template for modeling.^11^ To date, the majority of computational studies involving hepatic OATPs have been ligand-based, including different QSAR models,^12−18^ proteochemometric models,^12,19^ ligand-based pharmacophore models,^20^ and substructure analyses.^15^

**Figure 1:**
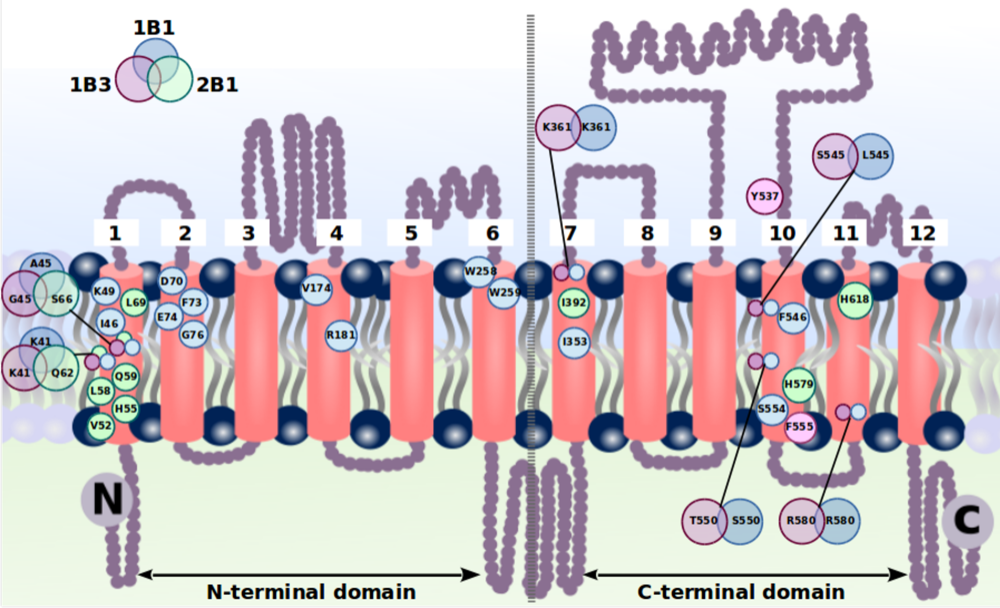
Schematic overview of relevant amino acid residues reported in the literature for the transport function or specificity of OATP1B1 (blue), OATP1B3 (magenta), and OATP2B1 (green).

OATPs are glycoproteins containing 643–722 amino acids. Hydropathy analysis shows 12 transmembrane helices (TMHs) interconnected by intracellular (IC) and extracellular (EC) loops,^21^ corresponding to the major facilitator superfamily (MFS) fold.^22^ MFS proteins contain multiple binding sites capable of recognizing structurally unrelated compounds. A large EC loop between TMH9 and TMH10 contains 11 cysteine residues which form disulfide bonds, resembling the Kazal-type domain of serine protease inhibitors (see Figure 1).^23^ Other important structural features are the N-glycosylation sites in the EC loops 2 and 5,^4^ phosphorylation sites at the N- and C-terminus,^24^ and the consensus sequence region spanning EC loop 3 and TMH6.^25^ The first structural models for OATP1B3 and OATP2B1 date back to 2005.^22^ On the basis of the comparison of OATP1B3 and OATP2B1 structures, ARG181 was suggested to contribute to OATP1B substrate specificity, while HIS579 was suggested as a structural determinant conferring OATP2B1 activity. Mandery et al.^26^ published newer structural models for OATP1B3 and OATP2B1 in 2011. Comparative analysis revealed that LYS361 and LYS399 are highly conserved across the OATP superfamily of membrane transporters, where LYS361 is pointing toward the translocation pore.^26^ In another study, mutagenesis experiments supplemented by structural model generation showed that several residues at THM2 of OATP1B1 (ASP70, PHE73, GLU74, GLY76) are implicated in transport function.^27^ Moreover, two distinct binding sites (low- and high-affinity binding site) for estrone-3-sulfate (E-3-S) were identified in that study. Another structure-based modeling study combined with alanine-scanning experiments identified the importance of TMH11 for OATP1B1-mediated ligand uptake.^28^ Further, Glaeser et al. used structure-based modeling supported by experimental validation to show the importance of a positive charge at position 41 and 580 for OATP1B3 transport function.^29^ Gui and Hagenbuch created a 3D structure of OATP1B3 and identified several crucial residues at TMH10 (TYR537, SER545, and THR550), which were subsequently analyzed via site-directed mutagenesis. In a recent study by Khuri et al., a combination of structure-based modeling and machine learning approaches was used to identify novel OATP2B1 inhibitors.^30^

An illustrative depiction of the overall protein organization into 12 TMHs and several IC and EC loops with an overview of amino acid residues with an experimentally confirmed involvement in ligand binding or transporter function is provided in Figure 1. Corresponding references are given in Table S1.

In this Article, we present an integrative computational pipeline for retrieving high quality structural models for OATP1B1, OATP1B3, and OATP2B1. The models were used to elucidate binding modes for steroid analogs highlighting commonalities and differences between the three transporters. To the best of our knowledge, this is the first comparative structure-based modeling study including all three hepatic OATPs.

Molecular determinants contributing to OATP-steroid interactions (and selectivity) have been analyzed in detail in our previous paper.^15^ Here, we are focusing on structural determinants as revealed by molecular docking to the developed transporter protein models, revealing distinct binding sites and ligand-transporter interactions. The computational findings presented herein have been validated retrospectively by comparison to published mutagenesis data and known single nucleotide polymorphisms. Structural models generated herein can be leveraged for, for example, virtual screening purposes to identify novel OATP interacting compounds. An in-house data set of new 13α-estrone derivatives with measured IC_50_ values on OATP1B1, OATP1B3, and OATP2B1 has been used in order to augment the compound data set from public sources with compounds showing a tendency toward higher affinity for OATP2B1 (which is to date the least studied transporter of the three hepatic OATPs).^31^ A special focus of the developed pipeline is ensemble docking. Inclusion of protein conformational flexibility is expected to increase confidence of the applicability of the structural models for docking. The application of ensemble docking in this study was motivated by the assumption that selectivity of SLC transporters might not be exclusively modulated by sequence variability but also by differences in conformational flexibility. Therefore, the final structural models were prioritized according to their ability to enrich known active compounds among a pool of known inactive compounds and decoys. To create a representative subset of OATP conformations, normal-mode analysis (NMA) was applied for available MFS structures to detect soft modes of motion which cover conformational diversity of MFS transporters (so-called “signature dynamics”).^32^ The pipeline presented here is versatile enough to be deployed for other protein targets of interest and was exclusively built upon freely available tools and software (pGenThreader,^33^ Modeler,^34^ ProDy,^35^ PHENIX,^36^ GROMACS,^37^ AutoDock Vina,^38^ PyMol,^39^ KNIME,^40^ Open Babel^41^), which enables full adaptability and reusability.

## Materials and Methods

All data, code, workflows, and models used or created in this study are available from an open GitHub repository: https://github.com/AlzbetaTuerkova/EnsembleDocking.

### Comparative Modeling of Hepatic OATPs

Structural templates were detected by the fold-recognition tool pGenThreader^33^ (default settings).

The fucose transporter in an outward-open conformation (FucP, PDB ID 3o7q, 3.14 Å resolution)^42^ possessing major facilitator superfamily (MFS) fold was identified as a high-quality template for OATP1B1 (*p*-value ≤ 0.0001, prediction score 75, 14.5% sequence identity), OATP1B3 *(p*-value ≤ 0.0001, prediction score 75, 15.6% sequence identity), and OATP2B1 (*p*-value ≤ 0.0001, prediction score 93, 15.2% sequence identity), respectively (see Table S2). In addition to a high prediction score for all the three transporters, the FucP template was selected because of its reasonable crystal structure resolution (3.14 Å), and an outward-open conformational state, which appears advantageous for studying ligand recognition.

PROMALS3D was used to generate multiple structure-to-sequence alignments between FucP and human OATP structures.^43^ The generated alignment was subjected to manual adjustments. Pairwise template-to-sequence alignments are available in the GitHub repository (https://github.com/AlzbetaTuerkova/EnsembleDocking; OATP1B1-FucP, OATP1B3-FucP, and OATP2B1-FucP). Because of the lack of structural templates for extra- and intracellular domains, our models cover the transmembrane region only. Amino acid residue numbers in the FucP template used for comparative modeling are the following: 22–56, 60–114, 115–177, 200–239, 242–290, 292–409, and 412–434. Corresponding regions in OATP1B1, OATP1B3, and OATP2B1 are listed in Table S3.

Enrichment docking into an ensemble of OATP conformations was conducted to prioritize the best model per transporter. Multiple OATP structures with various degrees of global (i.e., backbone conformer) and local (i.e., side-chain rotamer) flexibility were modeled. A similar strategy to the one applied by Carlsson et al.^44^ was adopted by performing NMA on the template structure. The modeling protocol introduced by Carlsson et al. has been expanded to perform more rigorous sampling of the protein conformational space. First, anisotropic network models (ANM) were calculated for all the available experimental structures possessing MFS fold to identify dominant motions within the whole protein family (so-called “signature dynamics”, for details see the Signature Dynamics of Major Facilitator Superfamily Proteins section). Second, alternate conformations for FucP were calculated by including the implicit membrane model (see the Conformational Sampling of the FucP Template section). NMA calculations were performed by using the ProDy software (freely available at http://prody.csb.pitt.edu/).^35^

#### Signature Dynamics of Major Facilitator Superfamily Proteins

The FucP template (PDB ID: 3o7q) was selected as a reference query for the retrieval of structurally analogous proteins by using the Dali server.^45^ In total, 92 protein structures with a shared fold were identified in the Protein Data Bank (PDB). Sequence identity between the structural analogs and the FucP structure was set to 10% to reduce the large pool of detected protein structures to a manageable amount. After data reduction, 45 PDB structures were retained in the structural ensemble. Twenty Gaussian Network Models (GNMs) modes per every protein structure were calculated at Cα carbon resolution. Calculated GNMs were analyzed with respect to commonalities and differences in the mode shapes, shared covariance between residues, cross-correlations, as well as mean square fluctuations. In addition, the similarity of protein structures in the structural ensemble was evaluated based on sequence (Hamming distance), 3D structure (RMSD), and their intrinsic dynamics (arccosine function of the covariance overlap). Specifically, the lowest frequency mode for each protein structure was used for this comparative analysis.

#### Conformational Sampling of the FucP Template

In this study, an implicit membrane model was incorporated into the ANM calculations.^46^ The restoring force for any protein displacement was set to be 16-times greater in the *x*- or *y*-direction than in the *z*-direction. The scaling factor was applied to preferentially restrain radial motions. Such defined restraints aim to mimic the constraints imposed by the membrane on the conformational dynamics of membrane proteins. Boundaries for the implicit membrane effect have been set to a distance of ±15.35 Å from the membrane core, as predicted by the OPM server.^47^

To prevent nonphysical distortions or bond stretching, individual residues were coarse-grained into predefined rigid blocks.^48^ Here, an assignment of residues into rigid blocks was done based on the hydrogen-bond estimation (DSSP) algorithm.^49^ Rigid block decomposition led to 135 blocks.^48^ One thousand alternate conformations were sampled along the two lowest frequency modes. In the next step, sampled conformers were refined by performing energy minimization in GROMACS 5.1.4,^37^ using the steepest descent algorithm in the AMBER99SB-ILDN force field.^50^ The convergence criterion was set to a maximum force <100.0 kJ/mol/nm. To reduce the large pool of conformational ensembles while preserving variance, only conformations with a cutoff distance of 3 Å from the average were kept resulting in 20 distinct conformers used for OATP structural modeling.

#### Construction of OATP Structural Models in Different Conformations

On the basis of the 20 FucP template conformers, 60 distinct models per transporter were calculated, following these consecutive steps:

(1) 20 different template conformers with an average RMSD of 3 Å were selected from the template conformational ensemble (counting 1000 conformers in total, see previous section).
(2) 100 comparative models per distinct template conformer were generated resulting in 2000 different models.
(3) N- and C- termini in helix breaks were acetylated (shortcut “ACE” in Modeler 9.17) and methylamidated (shortcut “CT3” in Modeler 9.17). Energy minimization of the comparative models was performed in GROMACS using the same settings as described in section 2.1.2.
(4) Minimized models were ranked on the basis of the MolProbity^51^ score calculated by using the PHENIX software.^36^

Validation analysis within the MolProbity tool consists of four consecutive steps: (a) Addition of hydrogen atoms. Asn/Gln/His flips are automatically corrected and −OH, −SH, and −NH_3_ groups are rotationally optimized. (b) All-atom contact analysis by probing the amount of overlap between the nonbonded atoms. The “clashscore” generated via contact analysis corresponds to the number of significant clashes (non-H-bond atom overlap > 0.4) per 1000 atoms. (c) Ramachandran and rotamer analyses of backbone and side-chains, respectively. (d) Covalent-geometry analysis by checking the outliers of backbone bond-lengths and bond-angles. The final MolProbity score unites all individual quality metrics into a single value. The MolProbity score was calculated for all 2000 models to filter out low-quality models.

Only three top-ranked rotamers per distinct conformer were retained for enrichment docking, resulting in 60 distinct models per transporter that were used for enrichment docking.

### Retrieving Ligand–Protein Interactions by Molecular Docking

#### Preparation of the Docking Library

Compounds with measured bioactivities (*K*_m_, IC_50_, *K*_i_, and EC_50_ bioactivity end points or percentage inhibition values) on OATP1B1 (*n* = 440 compounds), OATP1B3 (*n* = 936 compounds), or OATP2B1 (*n* = 173 compounds) were collected from the open domain by utilizing a KNIME workflow, which collects data from five independent data sources as described in Tuerkova et al.^15^ In addition, compounds from two of our recently published papers reporting pharmacological measurements for novel 13α-estrone derivatives were included.^52,53^ Experimental measurements for 16 13α-estrone derivatives have been extended in this study in order to report activity on all three transporters. IC_50_ values were calculated based on transport inhibition measurements (see below) by Hill1 fit, using the Origin Pro8.6 software (OriginLab Corporation, Northampton, MA, US).

13α-Estrone derivatives characterized in this study possess two major variations: (1) phosphorylation and (2) halogenation at either the R-2 or R-4 position of the steroidal core scaffold. The *R*3̅ position in the in-house data set is composed of a hydroxyl, methoxy, or benzyloxy moiety (see Table 1).

**Table 1:**
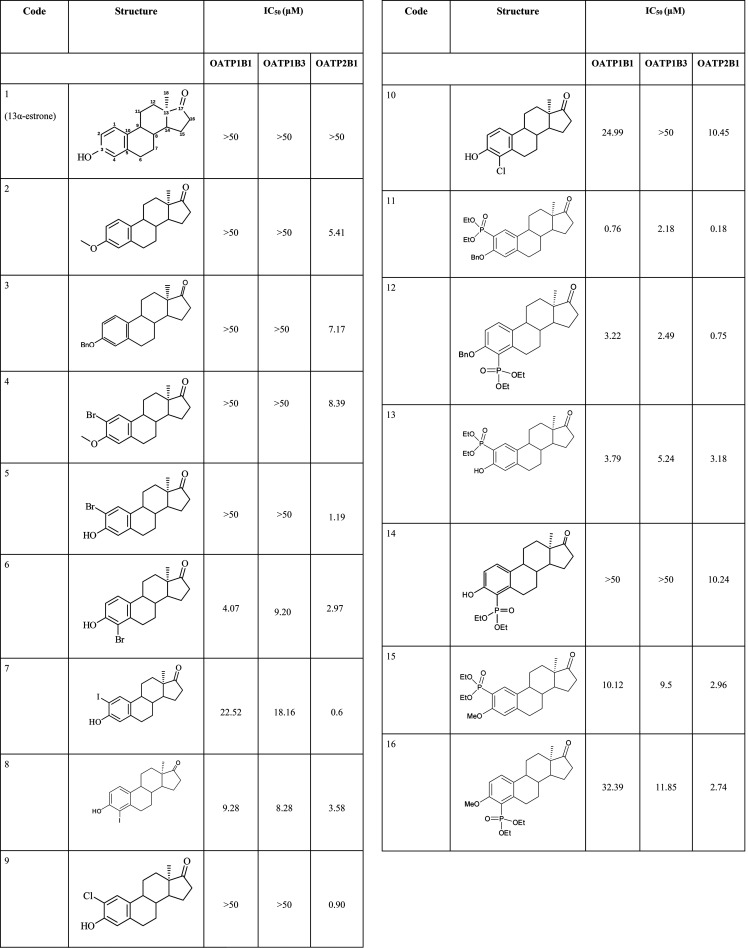
In-House Data Set of Steroid Analogs Used to Characterize Binding Sites of OATP1B1, OATP1B3, and OATP2B1^a^

a Chemical structures and bioactivity values (IC_50_ values in μM measured for the respective transporter) are given.

For the whole data set (public and in-house), frequencies of variations at different R-group positions (Figure S1) are in accordance with our previously published study on OATP ligand profiles.^15^

For the first two rounds of docking, for extracting the best protein model for each transporter, all compounds with bioactivity measurements were used—independent of their core molecular scaffold. The only exception was the in-house data set of 13α-estrone derivatives, which was used as a validation set. By including compounds with diverse core scaffolds, an unbiased selection of the “best” model (according to ligand enrichment) can be guaranteed. It has to be noted that this training data set did only contain a few compounds with a steroid scaffold for OATP1B1 (7 compounds) and OATP1B3 (4 compounds), but no steroidal structure for OATP2B1, which makes a bias of the model selection toward structures that better accommodate steroids less likely (especially in case of OATP2B1 this bias is impossible).

To obtain the recommended ratio of actives:inactives(/decoys) of 1:36, the data sets were enriched by decoys from DUD-E.^54^ A compound was defined as active if the bioactivity value was ≤1 μM in case of OATP1B1 and OATP1B3 and ≤5 μM in case of OATP2B1 (due to a smaller amount of data) and inactive if the activity value was >10 μM. Compounds with bioactivities falling into the region between >1 or >5 and ≤10 μM were excluded from the data set. If a compound occurred in different protonation states at pH 7.0 (±2.0), each protomer was considered as a separate compound for docking. The resulting docking library consisted of 57 actives, 917 inactives, and 1223 decoys for OATP1B1; 25 actives, 900 inactives, and no decoys for OATP1B3; and 12 actives, 153 inactives, and 279 decoys in case of OATP2B1. Ligand conformers were generated by the LigPrep tool in Maestro (version 19–1;^38^ OPLS3e force field). Ionization states were generated at target pH 7.0 ± 2.0 (Epik algorithm in Maestro).

#### Enrichment Docking for Model Prioritization

Compounds were docked into the top 60 models per transporter by using the program Autodock Vina 1.1.2.^38^ Exhaustiveness of the global search was set to 10. Ligand enrichment was calculated in *R*3̅.4.2 (available at https://www.r-project.org/). The area under the curve (AUC) and the enrichment factor (EF) at the top 1% of the data set (EF 1%) was calculated as a metric for ligand enrichment. In the first round of docking calculations, the entire transmembrane region (encompassing all 12 TMHs) was defined as a putative binding site. Models were ranked on the basis of their AUC values. Five top-ranked models per transporter were retained for further inspection. AUC values for the preselected models can be found in Table S4. For performing the second round of docking calculations, the putative protein binding site was further restricted. Specifically, the contact surface area between the active compounds docked in the first round was calculated, and the calculated region was used as search space in the second round of enrichment docking calculations. The procedure is visualized in Figure S2. The top final model was selected on the basis of the ranking of both AUC and EF 1% of the data set.

**Figure 2:**
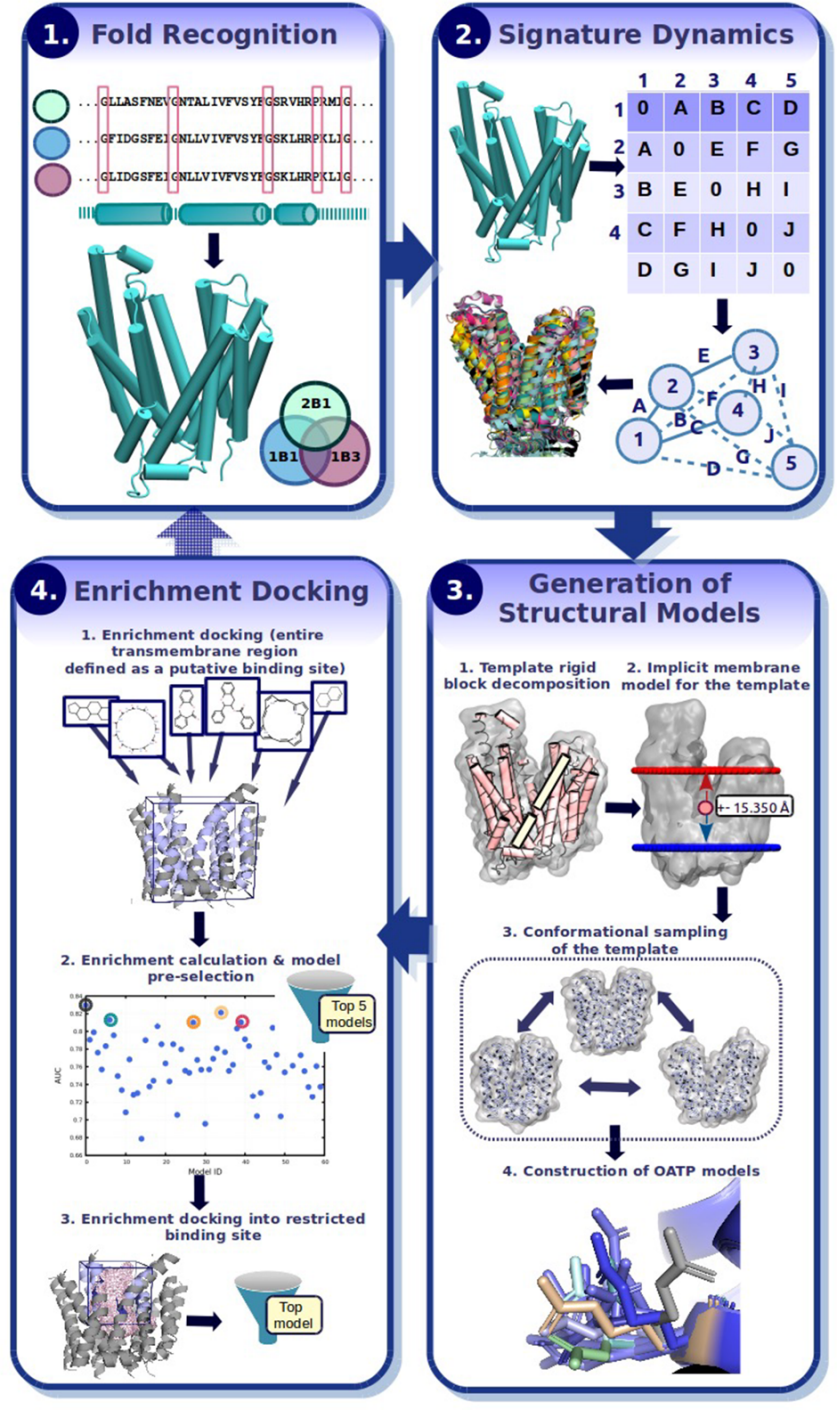
Major steps applied to retrieve structural models: In step 1, a fold recognition algorithm (here: pGenThreader) is used to identify the protein fold from the target sequences. Step 2 involves alignment of available protein structures possessing the desired fold. NMA is performed to identify conserved motions shared across the proteins with the same fold (here, called signature dynamics). In step 3, structural models (here, OATP1B1/OATP1B3/OATP2B1) are generated via a multistep procedure; First, the template structure (here, fucose transporter) is decomposed into individual rigid blocks via the hydrogen bond estimation algorithm (DSSP). Next, a scaling factor is applied to prioritize motions in the radial direction, which mimics the membrane environment (hence, implicit membrane model). Alternate conformers of the template structure are sampled via NMA. Structural models (here, OATP1B1/OATP1B3/OATP2B1) are subsequently built on the basis of different template conformations. For each template conformer, 20 structural models possessing 20 different side-chain rotamers are calculated. Enrichment docking (step 4) is performed in two consecutive steps: First, the entire transmembrane region is defined as a putative binding site (as indicated by the cube). Known active ligands and inactives/decoys are docked and the models are subsequently ranked on the basis of their AUC values. The top five models are retained. The contact surface area accommodating known actives from the first round of docking is calculated (visualized as pink mesh) and further used to restrict the search space for the second round of enrichment docking calculations. After the enrichment, docking is repeated into the top five models, and the best model is prioritized on the basis of its AUC value. In case no significant difference between AUC values was observed, the EF 1% was used as an additional metric to prioritize models.

The stepwise computational procedure for structural model generation is depicted in Figure 2.

#### Molecular Docking of Steroid Analogs

The 16 13α-estrone derivatives with in-house measured activity on OATP1B1, OATP1B3, and OATP2B1 used in this study are listed in Table 1. Supplementary File S4 in the GitHub repository (https://github.com/AlzbetaTuerkova/EnsembleDocking) lists steroidal compounds retrieved from public data sources along with their measured bioactivities (17 compounds). Correct stereochemistry of the steroidal nucleus was verified by comparing to experimentally resolved steroids in the Protein Data Bank (PDB) which were obtained via RESTful web services in KNIME^40^ (analogous to previously published work by our group).^55^

Autodock Vina 1.1.2.^38^ was used to dock steroid analogs. Ten binding modes were sampled. Exhaustiveness of the search was set to 20. To map possible interaction sites for steroid analogs, the entire transmembrane region was defined as a putative binding site.

Docked poses were analyzed via hierarchical pose clustering as follows: (1) A docked ligand structure was reduced to its core Murcko scaffold^56^ (saved in pdbqt format), (2) a pdbqt file with a core scaffold was converted into a mol file format using Open Babel 2.4.1.,^41^ (3) the maximum common substructure (MCS, here [#6]1-,:[#6]-,:[#6]-,=,:[#6]2-,:[#6](-,:[#6]-,=,:1)-[#6]1-[#6]-,=[#6]-[#6]3(-[#6](-[#6]-1-[#6]-[#6]-2)-[#6]-[#6]-[#6]-3)-[#6] in SMARTS, see Figure S1) for all retrieved core scaffolds was calculated (using the FindMCS functionality in RDKit; bond order kept flexible) by using an in-house script (available as Supplementary File S5 in the GitHub repository (https://github.com/AlzbetaTuerkova/EnsembleDocking)), (4) output coordinates were saved in xyz format and converted back to pdbqt format using OpenBabel 2.4.1. (5) MCSs were loaded into PyMOL,^39^ and (7) the agglomerative hierarchical clustering algorithm within the PyDRA plugin was used to calculate average distances (distance cutoff set to 2 Å).

Cluster analysis was performed in KNIME 4.1.2.^40^ Compounds were analyzed by calculating protein–ligand interaction fingerprints (PLIFs) in MOE.^57^ H-donor (cutoff 0.5–1.5 [kcal/mol]), H-acceptor (cutoff 0.5–1.5 [kcal/mol]), ionic attraction (cutoff 0.5–3.5 [kcal/mol]), metal ligation (cutoff 0.5–3.5[kcal/mol]), and arene attraction (cutoff 0.5–1.0 [kcal/mol]), were defined as distinct interaction types used in the calculation. The pocket volume was calculated via the open-source POVME binding pocket analysis software.^58^ The radius of gyration was calculated by using the gyradius functionality within the Psico module (a PyMOL extension).

### Transport Inhibition Experiments for 13α-Estrone Derivatives

13α-Estrone derivatives were synthesized previously, as described in Jójárt et al.^52^ and Bacsa et al.,^59^ 20 mM stocks in DMSO were stored for further usage at −20 °C. A431 cells overexpressing OATPs, OATP1B1, OATP1B3, or OATP2B1 or mock transfected controls were generated previously.^60^ A431 cells were maintained in DMEM medium (Thermo Fischer Scientific, Waltham, MA, US) supplemented with 10% fetal bovine serum, 2 mM l-glutamine, 100 units/mL penicillin, and 100 μg/mL streptomycin at 37 °C with 5% CO_2_. The interaction of 13α-estrone derivatives with OATPs, 1B1, 1B3, and 2B1 was measured in A431 cells overexpressing the given OATP using the previously identified OATP1B and OATP2B1 substrate pyranine (8-hydroxypyrene-1,3,6-trisulfonic acid trisodium salt, Sigma, Merck, Hungary). The uptake of pyranine was measured on microplates based on the method developed by us previously.^60,61^

Briefly, 1 day prior to the uptake measurement cells (8 × 10^4^ cells/well in 200 μL DMEM) were seeded on 96-well plates. On the following day, the medium was removed, and the cells were washed three times with 200 μL of phosphate-buffered saline (PBS, pH 7.4) and preincubated with 50 μL of uptake buffer (125 mM NaCl, 4.8 mM KCl, 1.2 mM CaCl_2_, 1.2 mM KH_2_PO_4_, 12 mM MgSO_4_, 25 mM MES (2-(*N*-morpholino)ethanesulfonic acid, and 5.6 mM glucose, pH 5.5) with or without increasing concentrations of the tested compounds. The reaction was started by the addition of 50 μL of uptake buffer containing pyranine in a final concentration of 10 μM (OATP1B1) or 20 μM (OATP1B3 and OATP2B1). Cells were incubated with the dye at 37 °C for 15 min (OATP1B1 and OATP2B1) or 30 min (OATP1B3), after which the supernatant was removed, and the cells were washed three times with 200 μL of ice-cold PBS. Fluorescence (in 200 μL of PBS/well) was determined in an Enspire plate reader (PerkinElmer, Waltham, MA, Ex/Em = 403/517 nm). OATP-dependent transport was calculated by extracting fluorescence measured in mock transfected cells. Transport activity was calculated based on the fluorescence signal in the absence (100%) of the tested compounds. Experiments were repeated at least three times on cells deriving from different passages.

IC_50_ values were calculated by Hill1 fit, using the (OriginLab Corporation, Northampton, MA, US).

## Results and Discussion

### Insights from Conformational Sampling of Experimentally Resolved MFS Structures and Ensemble Docking into OATP Structural Models

Biologically relevant motions, such as protein conformational changes happen at time scales in the range of micro- to milliseconds or even seconds and, therefore, generally cannot be studied by classical molecular dynamics (MD) simulations. By using normal-mode analysis (NMA), functional protein motions can be captured by global (soft) modes, which represent collective motions of entire protein (sub)domains. In this study, the motivation for inclusion of NMA was 2-fold: (1) To compare normal modes for available structures of the major facilitator superfamily (MFS) members (45 structures) from the Protein Data Bank (PDB) and, thus, identify functionally important protein motions and (2) to sample alternate template conformers.

In the former case (1), our intention was to explore how intrinsic protein dynamics might diverge across a protein family with a shared fold (so-called signature dynamics). Global fluctuations shared across the MFS proteins might deliver useful insights into the transport mechanism. In the latter case (2), we incorporated the knowledge about MFS dynamics from step 1 into our ensemble docking strategy in such a way that anisotropic network models (ANMs) for the selected template (here, FucP; PDB ID 3o7q) were calculated.

#### Signature Dynamics of MFS Transporters

**Figure 3:**
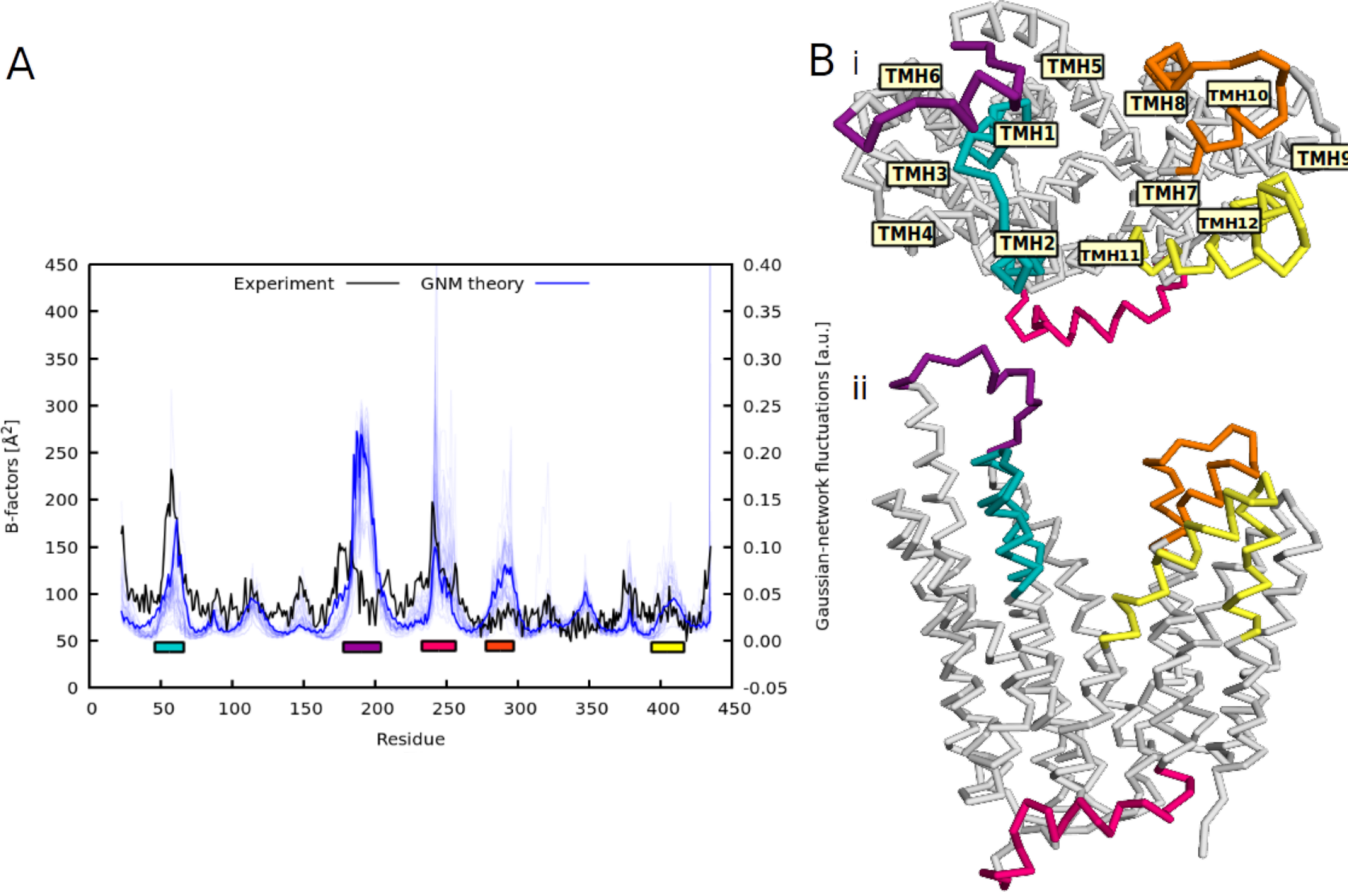
(A) Root mean square fluctuations of the selected MFS proteins (*n* = 45) derived from GNMs (blue curves) and from the X-ray structure of FucP transporter (PDB ID 3o7q, black curve): The experimental root-mean-square fluctuations are indicated in A^2^ units, while the theoretical calculations are given in arbitrary units. The regions of fluctuations discussed in the text are marked by colors. The coloring corresponds to specific regions in the MFS structure, as shown in panel B: (i) top view with transmembrane helix numbering and (ii) side view.

To assess the feasibility of the elastic network models for exploring the intrinsic dynamics of MFS proteins, root-mean-square fluctuations (RMSF) derived from NMA can be compared with crystallographic B-factors for experimentally determined structures. In this study, the crystal structure of FucP transporter (PDB ID 3o7q) was taken as a reference to compare NMA-based fluctuations of α-carbons with the B-factors from X-ray crystallography (Figure 3). A RMSF profile of the five softest modes (mode 1–mode 5) of 45 proteins with MFS fold from PDB (Figure 3A and Figure S3) exhibited significant fluctuations in distinct regions (based on residue numbering): 45–68 (TMH1-2), 182–195 (EC), 240–260 (IC), 280–302 (TMH7), and 392–420 (TMH11; see colored regions in Figure 3A).

The high peak observed for the region 182–195 for the GNM-based fluctuations is caused by a significantly extended TMH1 reaching to the EC region for some transporters (such as human GluT3 transporter; PDB IDs 4zwb, 4zwc, 4zw9).^62^ Loop regions possess lower numbers of inter-residue contacts in the elastic network, which leads to higher flexibility in these regions. These findings are consistent with other network models for MFS transporters available in the literature.^63^ Similarly, large fluctuations in region 240–260 (corresponding to the cytoplasmic region) are likely caused by the presence or absence of specific IC domains, such as the YAM domain of *E. coli* transporter YarjR (PDB ID 3wdo)^64,65^ or the IC helical (ICH) domain consisting of three to four helices in sugar transporters.^66,67^

Because of the structural ambiguity of extra- and intracellular regions, structural models of hepatic OATPs generated herein cover transmembrane regions only. Specifically, our primary aim was to unravel binding modes for steroid analogs which are chemically closely related to endogenous substrates of these transporters (such as DHEA). Interestingly, mode 1 and mode 2 of NMA show mutual (out-of-plane) shifts in the upper part of TMH1 and TMH2 (region 45–68). Fluctuation values of this region are in a good agreement with experimental fluctuations for the FucP transporter structure (Figure 3A), and they are located inside the transmembrane core thus they could have an impact on ligand accessibility and binding. The other (albeit less pronounced) TMH motions are located in the upper part of TMH7 (280–302) and TMH11 (392–420). Again, the fluctuations of these regions share corresponding mode shapes with B-factors from experiments (Figure 3A). These findings prompted us to generate alternate conformations of the FucP template (PDB ID 3o7q) along mode 1 and mode 2, reflecting fluctuations in TMH1, TMH2, TMH7, and TMH11. Specific settings for conformational sampling of the template (including the implicit membrane model) can be found in the methodological section (Conformational Sampling of the FucP Template section).

By reordering sequence- and structure-based matrices according to the “dynamics-based” similarities, a cluster of analogous proteins (*n* = 16, Figure S4) was identified. Interestingly, some of the dynamically related proteins (PDB ID 4m64, 3wdo, 4gc0) were predicted by the pGenThreader algorithm as suitable templates to model hepatic OATPs (Table S5). These findings show that the structural templates individually predicted by fold recognition tools are related not only sequentially and structurally, but also dynamically, which increases the confidence in fold recognition methods for detecting valuable templates. For this study, however, no better suited template was detected by this method for modeling hepatic OATPs.

#### Ensemble Docking into OATP1B1, OATP1B3, and OATP2B1 Structural Models

Studying the ROC curves at EF1% for the top five models for OATP1B1 (AUC = 0.68–0.83, EF1% = 0.0–5.0%), OATP1B3 (AUC = 0.75–0.94, EF1% = 18.5–23.8%), and OATP2B1 (AUC = 0.50–0.70, EF1% = 0.0–6.04%), differences in the model performances become obvious (Figure S5B). The models’ abilities to separate highly actives from inactives/decoys performed best for the OATP1B3 models, despite a smaller data set of actives when compared to OATP1B1 (25 actives vs 57 actives). This phenomenon can be explained by the fact that in case of OATP1B1 docking 1223 decoys from DUD-E have been added to the set of measured inactives, whereas for OATP1B3 docking only measured inactives (*n* = 900) were used for ligand enrichment calculations. The ROC curves of the top OATP1B1 models are flatter since obviously the decoys are more often falsely classified as actives (false positives) than the measured inactives. The comparably least performing models were those for OATP2B1, which can be explained by the small overall compound set for docking (only 12 highly actives, 153 inactives, 279 decoys) and a (relatively) weaker cutoff for defining activity that was used in this case (≤5 μM).

Interestingly, a certain trend between AUC values and the radius of gyration of the OATP structural models was observed. An example is given in Figure S5A for OATP1B1 models. Similarly, an increased pocket volume of the translocation pore is related to an increase in AUC values. The five prioritized models for OATP1B1, OATP1B3, and OATP2B1, differ in terms of their 3D structure from the initial template conformation (average RMSD of 2.8 Å for OATP1B1, 3.1 Å for OATP1B3, and 3.2 Å for OATP2B1; Figure S5C). Specifically, correlated movements of TMH1 and TMH2 (out-of-plane motion), as well as the fluctuations in the upper part of TMH7 and TMH11 (opening the central cavity, possess the highest deviation from the initial template (up to 5.9 Å for TMH2 in OATP2B1). These observations indicate that the models might benefit not solely from the increased ligand accessibility (as evidenced by the increase of the radius of gyration and pocket volume compared to the initial template structure), but also from the specific directionality of TMH1 and TMH2, which in turn impacts geometry of the N-terminal domain. In addition, all three prioritized models for the different OATP transporters possess an equivalent protein conformational state (open-to-out state with an TMH1/2 out-of-plane shift, see Figure S5C). These findings suggest that OATP selectivity is likely driven by subtle variations in amino acid sequences rather than by significant differences of protein conformations of the three transporters.

**Figure 4:**
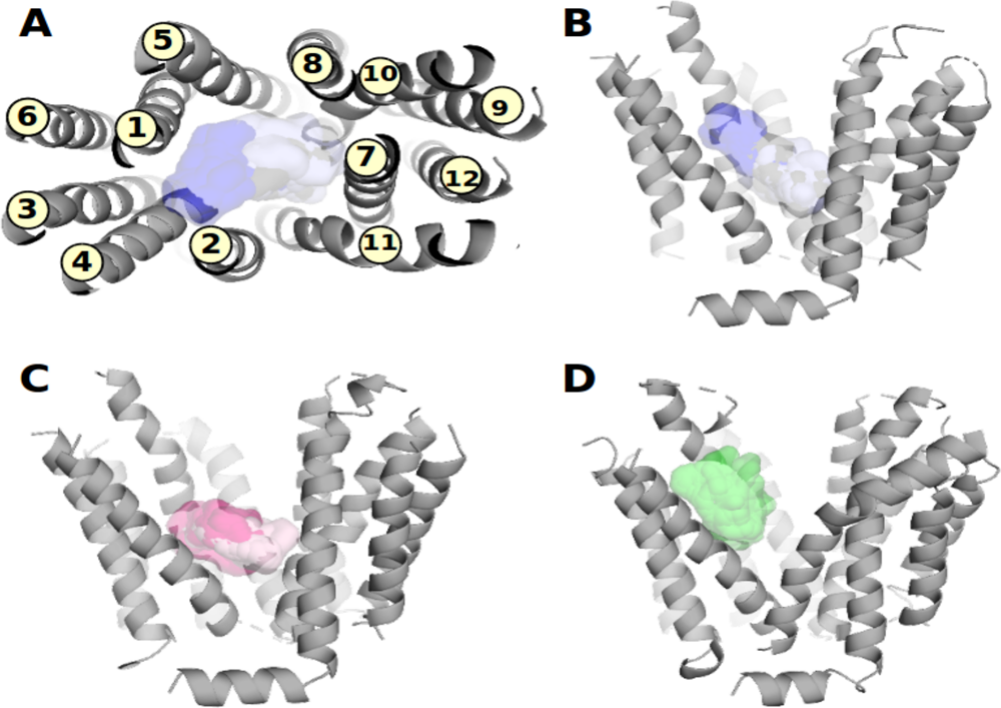
Sites of steroid binding for the enriched clusters of docking poses in the respective transporters (visualized as transparent van der Waals surfaces): (B) OATP1B1, (C) OATP1B3, and (D) OATP2B1. Enriched clusters are shown in different color grades. For OATP1B1, a top view of the protein with numbered TMHs is included for better orientation (A).

The final structural models used in this study are shown in the GitHub Repository (https://github.com/AlzbetaTuerkova/EnsembleDoc) and are depicted in Figure S6). An overview of amino acid residues spanning the different transmembrane regions in the three transporter models is given by the amino acid sequence alignment depicted in Table S3. Residues that are conserved across the three transporters are marked in this table.

### Shared and Distinct Interactions of Steroid Analogs with Hepatic OATPs

Analyzing common and distinct binding modes of the three related transporters can be carried out systematically by pose clustering and frequency analysis of transporter-ligand interactions. Cluster analysis yielded 15 distinct clusters for OATP1B1, 9 distinct clusters for OATP1B3, and 9 distinct clusters for OATP2B1, respectively (Tables S7–S9). Clusters per transporter were prioritized on the basis of both the number of poses per cluster (compounds may appear more than once in a single cluster) and the number of unique compounds per cluster. Filtering for clusters that possess more than 50% of the actives per respective transporter, three distinct clusters for OATP1B1 (85%, 65%, and 55% of unique compounds), two distinct clusters for OATP1B3 (100% and 83% of unique compounds), and one single cluster for OATP2B1 (94% of unique compounds) were retained for further investigations (the location of the respective clusters in the transporters is depicted in Figure 4).

Next, protein–ligand interaction fingerprint (PLIF) analysis gave insights into protein–ligand interactions which are shared across the three transporters and those that are specific for a certain transporter (Tables 2 and S9 and Figures S7–S9).

**Table 2:**
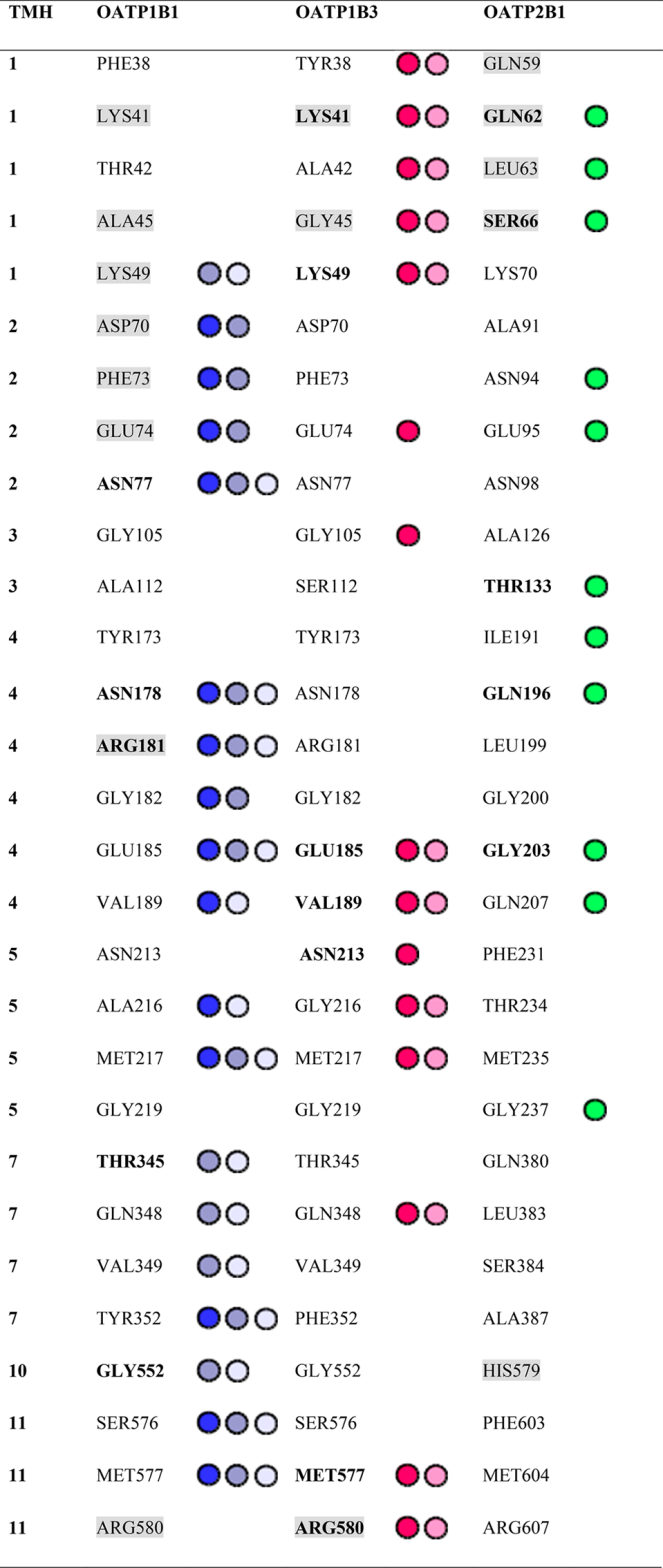
Key Amino Acid Residues Involved in Ligand Binding Considering Prioritized Clusters Only^a^

a Prioritized clusters are indicated by colored circles next to the residues: white, 1st enriched OATP1B1; gray, 2nd enriched OATP1B1; blue, 3rd enriched OATP1B1 cluster; dark pink, 1st enriched OATP1B3 cluster; light pink, 2nd enriched OATP1B3 cluster; green, top enriched OATP2B1 cluster. Residues that are reported in the literature to be important for transport activity are highlighted in gray color. Residues that appear to be involved in binding of steroid analogs with a higher frequency in this study (at least 10% of all poses or interaction partners for the respective transporter) are marked in bold font. TMH numbers are annotated in the left column.

Studying the top three ligand clusters in OATP1B1, it becomes obvious that many amino acids are shared (or overlapping) between the clusters (e.g., 8 out of 19 residues are shared among all three clusters, see Table 2). The two top-ranked clusters in OATP1B1 (accommodating 85% and 65% of the docked compounds, respectively) reach into the central cavity of the transporter, being enframed by TMH5, TMH7–8, TMH10–11. The third cluster (55% of docked compounds) is located closer to the N-terminal domain of the transporter and is lined by TMH1–5 (Figure S7).

In OATP1B3, the top-ranked cluster interacts with residues from TMH1–5, (and partly TMH7 and TMH11), similarly to the third cluster in OATP1B1. The second prioritized ligand cluster for OATP1B3, however, is located a bit closer to the central cavity, lined by TMH1, TMH2, TMH7, and TMH10–11. Here, even more residue interactions are shared among the two ligand clusters (14 out of 17), making a strict separation of the observed binding modes even more difficult for this transporter (Figure S8).

In contrast to OATP1B1 and OATP1B3, OATP2B1 ligand cluster analysis did only prioritize a single binding site near the N-terminus (interacting with residues from TMH1–5) with 94% of active ligands docked into this site (Figure S9).

#### Transmembrane Helices of Hepatic OATPs Stabilized by Salt Bridges

Across all three transporters, there is only a single conserved amino acid residue that was prioritized during steroid docking: GLU74 (in OATP1B1 and OATP1B3)/GLU95 (in OATP2B1) at TMH2 (Table 2 and Figure S10).

The importance of GLU74 and other residues at TMH2 (ASP70, PHE73, GLY76) for E-3-S transport by OATP1B1 has previously been confirmed by mutagenesis studies. Mutation of these residues to alanine led to a significant loss of E-3-S uptake activity. Li et al.^27^ have postulated the role of GLU74 for transport function to be mainly acting as a stabilizing factor for the binding site by formation of a salt bridge with a nearby positively charged amino acid.

In the generated protein models from this study, the formation of intramolecular salt bridges by GLU74/95 with a positively charged residue from a TMH lying opposite of TMH2 was consistently found in all the three transporters. In OATP1B1 and OATP2B1, we observed salt bridge formation of GLU74/95 with ARG580/607 on TMH11 (OATP1B1/OATP2B1) while in OATP1B3, the intramolecular interaction was formed with LYS49 on TMH1 instead (Figure S11).

In OATP1B1 and OATP1B3, ARG580 was identified as an important functional residue and postulated to be either involved in substrate binding or to be part of the translocation pathway.^29,68^ We did not observe direct steroid interactions with this positively charged residue in OATP1B1 and OATP2B1 in our study (which is obviously hindered by the formation of intramolecular interactions), but only in OATP1B3 (see Tables 2 and Table S9).

In OATP1B1, LYS41 (TMH1), and GLU185 (TMH4) are forming an additional stabilizing salt bridge (Figures S11 and 5A–E). These observations point to the importance of positively charged residues in the substrate translocation pore as also hypothesized by Meier-Abt et al.^22^

**Figure 5:**
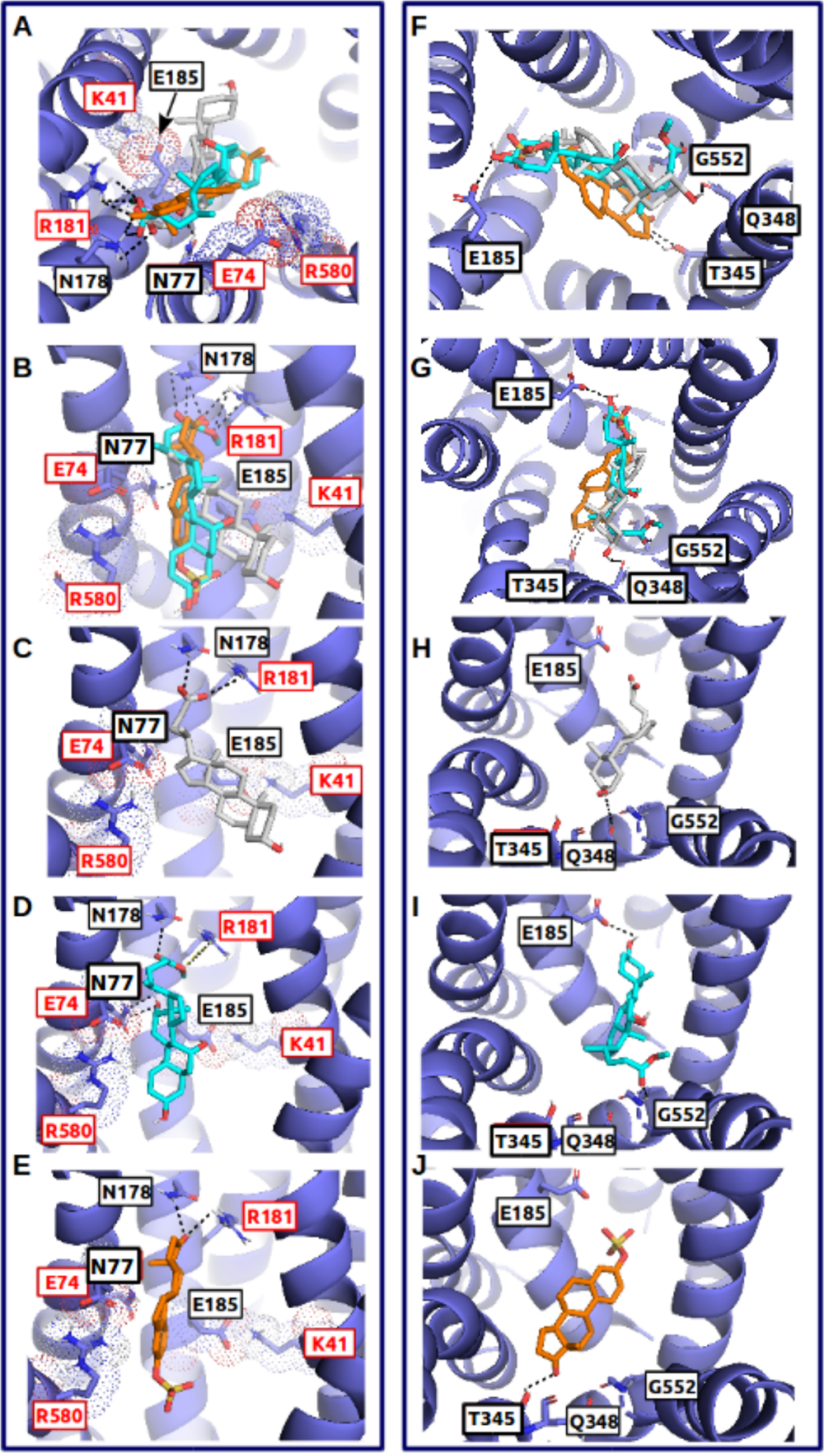
Ligand binding to the N-terminal binding site (left panel) and the C-terminal/central binding site (right panel) in OATP1B1: Several strong steroidal OATP1B1 inhibitors are shown in the top view (A and F) and side view (B and G), respectively. Panels C and H are showing lithocholate (light gray, median bioactivity value [μM] = 0.918)). Panels D and I are showing cholic acid methyl ester (cyan, median bioactivity value [μM] = 0.20). Panels E and J are showing E-3-S, which is known to be an OATP1B1 selective inhibitor (orange, median bioactivity value [μM] = 0.450). Hydrogen bonds are visualized via black dashed lines. The residues labeled in red were validated via mutagenesis studies published in the literature. Dotted surfaces around certain residues visualize van der Waals radii in order to highlight residues that are forming intramolecular salt bridges between protein residues (K41, E74, E185, R580).

It has to be emphasized that the observed differences in salt bridge formation between the transporters cannot be attributed to differences in amino acid residues of the salt bridge forming interaction partners. As seen from Table 2, all of the mentioned residues are conserved across all three transporters. It is tempting to speculate that the salt bridges formed in the respective final selected models are just reflecting one possible plausible intramolecular interaction state.

#### Commonalities and Differences of Steroid Analog Binding to OATP1B1 and OATP1B3

Overall, for steroid analogs with OATP1B1 activity, no prominent interactions (at least 10% of all interactions) with residues at TMH1, TMH5, and TMH11 occurred in our study, while for steroids with activity on OATP1B3, residues at TMH1 (LYS41, LYS49), TMH4 (GLU185, VAL189), TMH5 (ASN213), and TMH11 (MET577, ARG580) are among the main interacting partners (see Tables 2 and S9). In experimental studies, LYS41 and ARG580 have been shown to play a role in transport activity in both OATP1B1 and OATP1B3.^29,68,69^ OATP1B1 active steroids are mainly showing TMH2 (ASN77), TMH4 (ASN178 and ARG181), TMH7 (THR345) and TMH10 (GLY552) involvement in our study (see Tables 2 and Table S9). ARG181 was found to be a functionally important residue in OATP1B1, indicated by site-directed mutagenesis studies.^70^

**Figure 6:**
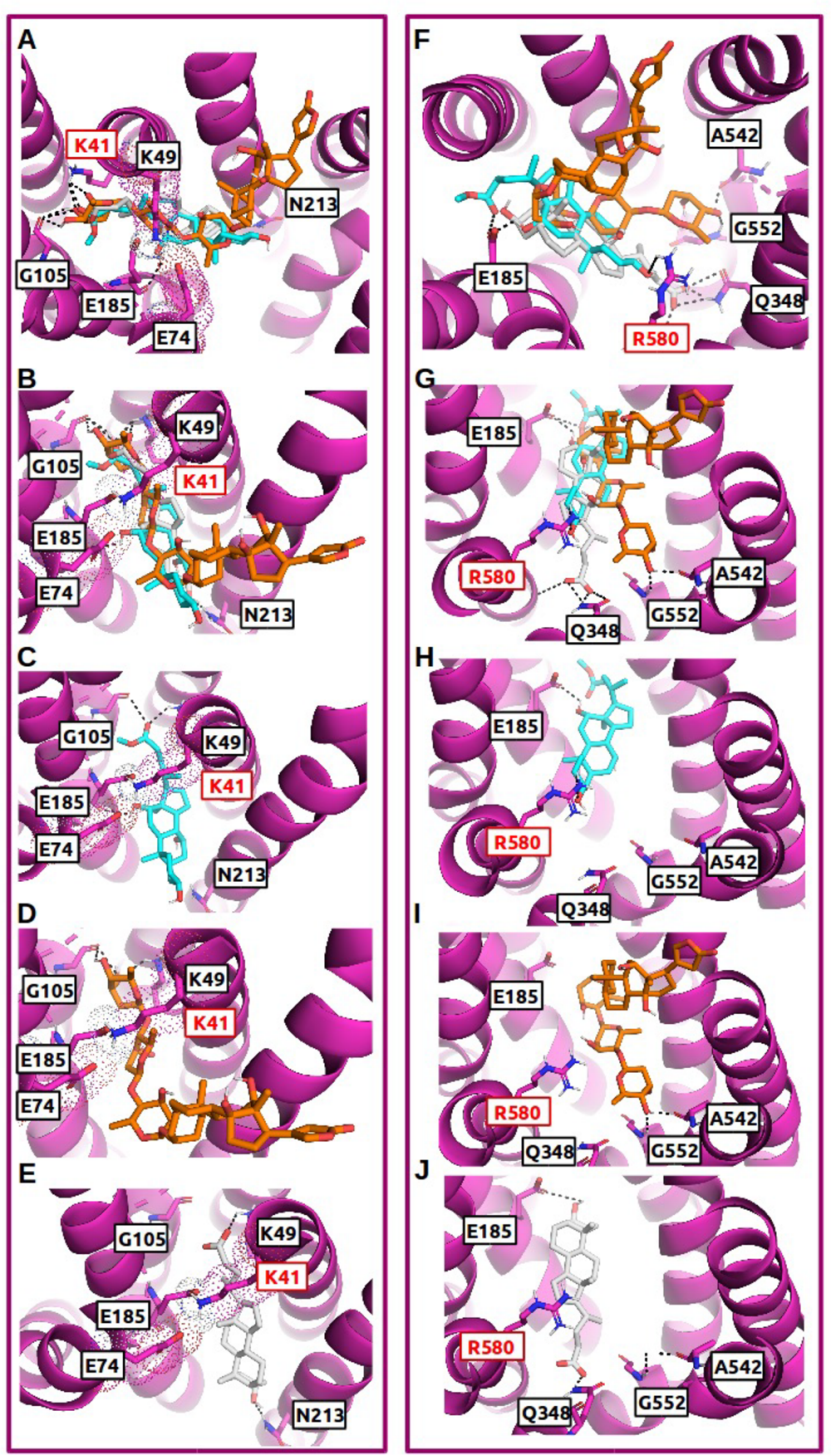
Ligand binding to the N-terminal binding site (left panel) and the C-terminal/central binding site (right panel) in OATP1B3: Several strong steroidal OATP1B3 inhibitors are shown in the top view (A and F) and side view (B and G), respectively. Panels C and H are showing cholic acid methyl ester (cyan, median bioactivity value [μM] = 0.13). Panels D and I are showing digoxin, which is known to be an OATP1B3 selective inhibitor (orange, median bioactivity value [μM] = 0.836). Panels E and J are showing lithocholate (light gray, median bioactivity value [μM] = 6.807). Hydrogen bonds are visualized via black dashed lines. Residues labeled in red were validated via mutagenesis studies published in the literature. Dotted surfaces around certain residues visualize van der Waals radii to highlight residues that are forming intramolecular salt bridges between protein residues (E74, K49).

Inspecting docking poses of several strong OATP1B1 inhibitors (E-3-S, lithocholate, and cholic acid methyl ester) in OATP1B1, we could observe a relatively consistent pattern of binding orientations in the central as well as in the N-terminal binding cavity. In general, the ligands appear to be vertically oriented in OATP1B1, whereas in OATP1B3 we observed a horizontal orientation of OATP1B3 binders (digoxin, lithocholate, and cholic acid methyl ester) and a less consistent binding pattern (Figures 5, 6, and S12). As pointed out in the previous paragraph slightly different orientations of dual OATP1B1/OATP1B3 inhibitors (e.g., lithocholate, cholic acid methyl ester, beclomethasone) in the analogous cavities of the two transporters are likely caused by the different intermolecular salt bridge formations that force the ligands into certain orientations due to differences in pocket accessibility. As seen from Figures 6A–E and S12D–E, the salt bridge LYS49-E74 (TMH1-TMH2) in OATP1B3 restricts the N-terminal pocket in a way that the horizontal orientation is favored.

Differences in the interactions with amino acid residues of dual OATP1B1 and OATP1B3 inhibitors (lithocholate, cholic acid methyl ester) are shown in Figures 5 and 6 for both the N-terminal and central binding cavities in both transporters, highlighting the above-mentioned main contributing residues.

To relate subtle differences of amino acid side chain nature and orientation in the binding pockets and their different accessibility for steroidal compounds in OATP1B1 and OATP1B3 to ligand selectivity, we examined poses of the two selective steroidal compounds E-3-S (OATP1B1 selective) and digoxin (OATP1B3 selective) in more detail.

We identified an interaction of E-3-S with Y352 (TMH7) as a potential reason for OATP1B1/OATP1B3 selectivity. The side chain hydroxyl group of Y352 interacts with E-3-S through hydrogen bond formation, which in turn leads to an orientation of E-3-S, where the ligand is deeply buried in a hydrophobic cavity (Figure S13). In OATP1B3 the hydrogen bond formation with TYR352 is disabled (PHE at the same position). The hydrophobic pocket forming residues include the nonconserved VAL349 in OATP1B1 (SER in OATP1B3) and the conserved ILE353. Interestingly, ILE353 is a known site of OATP1B1 single nucleotide polymorphism (ILE353THR) related to a decreased OATP1B1 transport activity. Other steroidal compounds also adopt a similar binding mode where TYR352 acts as a hydrogen bond donor/acceptor (in total a decent amount of 7% of all poses interact with this residue).

**Figure 7:**
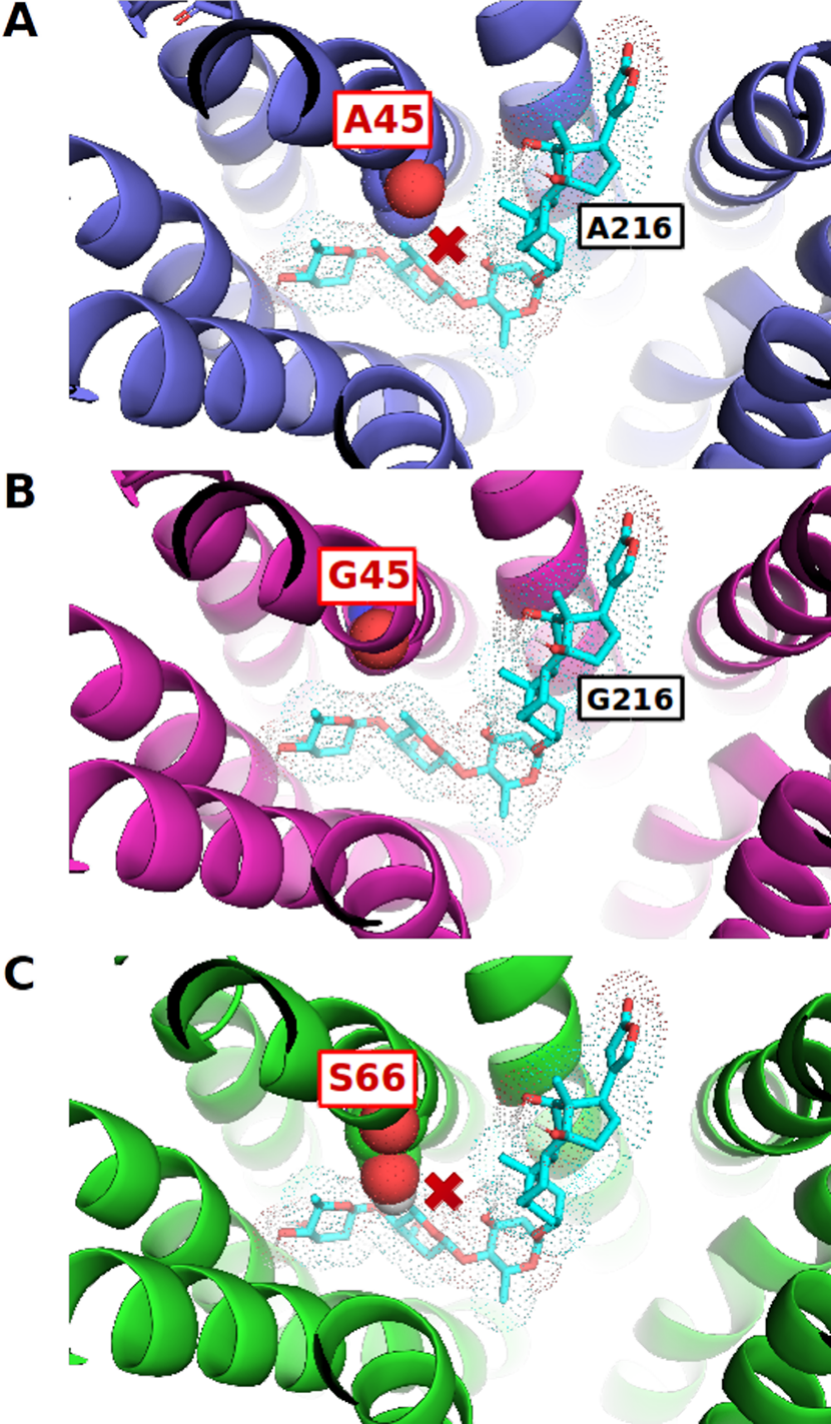
Digoxin helps explain OATP selectivity: Color code: docked compounds = cyan (carbon), red (oxygen), orange (phosphorus), OATP1B1 = blue, OATP1B3 = magenta, OATP2B1 = green. van der Waals radii of ALA45 (A), GLY45 (B), and SER66 (C) are visualized via van der Waals radius. Red cross indicated steric clash of a residue with the ligand. Poses are shown from the top (from the extracellular part). In addition, the position of ALA/GLY216 (OATP1B1/1B3 nonconserved residue) is depicted in this figure.

Digoxin is a cardiac glycoside with a chain of three sugars in position *R*3̅ of the steroidal ring, which is selectively recognized by OATP1B3. The sugar moiety is buried deeply in the N-terminal domain of OATP1B3 interacting with GLY105 (main chain interaction) and LYS41 (side chain interaction) via the formation of hydrogen bonds (Figure 6D). An equivalent pose in OATP1B1 was not retrieved by molecular docking. However, manually placing digoxin into OATP1B1 at the equivalent position, it becomes evident that ALA45 (which is a glycine in OATP1B1) would lead to a steric clash with the large sugar moiety (Figure 7).

ALA45/GLY45 at TMH1 has previously been reported and experimentally confirmed to be crucial for cholecystokinin-8 (CCK-8) transport by DeGorter at al.^71^ Through the loss of the methyl group by A45G mutation in OATP1B1, an increase of CCK-8 transport was observed. In our models, the pocket volume increases in case of OATP1B3 through this amino acid exchange (551 A^3^ for OATP1B3 compared to 510 A^3^ for OATP1B1 for our models), which leads to better pocket accessibility.

According to the current state of the art, we can, therefore, assume that differences in preferences for steroid analog binding to OATP1B1/OATP1B3 can be attributed to differences in pocket accessibility in the N-terminal part of the transporters (tentatively induced by nonconserved residues in TMH1), as well as to nonconserved amino acids in the central binding cavity (especially those located on TMH7).

#### Interactions of Steroid Analogs with OATP2B1

OATP2B1 only has a few residues in common with OATP1B1 and OATP1B3 when studying the docked interactions with steroidal compounds, which are all located in TMH1 and TMH2 (Table 2). This behavior was to be expected due to the remarkable difference in amino acid sequence identities between the OATP1B subfamily and OATP2B1 (identity only around 31%). Interestingly, among the shared interactions, those formed with residues at TMH1 (GLN62, LEU63, and SER66) are shared exclusively with OATP1B3 (corresponding to LYS41, ALA42, and GLY45), whereas some of the main interactions with residues at TMH2 (ASN94) are shared with OATP1B1 only (corresponding to PHE73). GLU95 (GLU74 in OATP1B1 and OATP1B3) is the only shared interacting residue among all three transporters (as discussed earlier).

The data set of 13α-estrone derivatives (16 compounds in total; see Table 1) served as a validation set for our procedure. 13α-estrones were chosen since steroids are highly enriched among OATP substrates, and furthermore the sterane core allows various modifications helping to reveal structure function relationship.

Here, we investigated the inhibitory activity of 13α-estrone derivatives using the previously validated general OATP1B1, OATP1B3, and OATP2B1 fluorescent substrate pyranine. In these measurements, A431 cell lines overexpressing one of the aforementioned OATPs were used and IC_50_ values for the compounds have been determined by measuring transport inhibition at increasing concentrations of the 13α-estrones. Since data for OATP2B1 is to date very sparse in the open domain, especially the new bioactivity measurements on OATP2B1 are a valuable source of information in order to better understand OATP2B1-ligand interactions and potential drivers for selectivity. The latter is in particularly supported by this new data set, since 5 of the 16 compounds are showing selectivity toward OATP2B1 (between at least 6-fold difference and 55-fold difference in activities toward OATP2B1 vs the other two transporters), and 3 additional compounds are showing preferential activity toward OATP2B1 (see Table 2).

**Figure 8:**
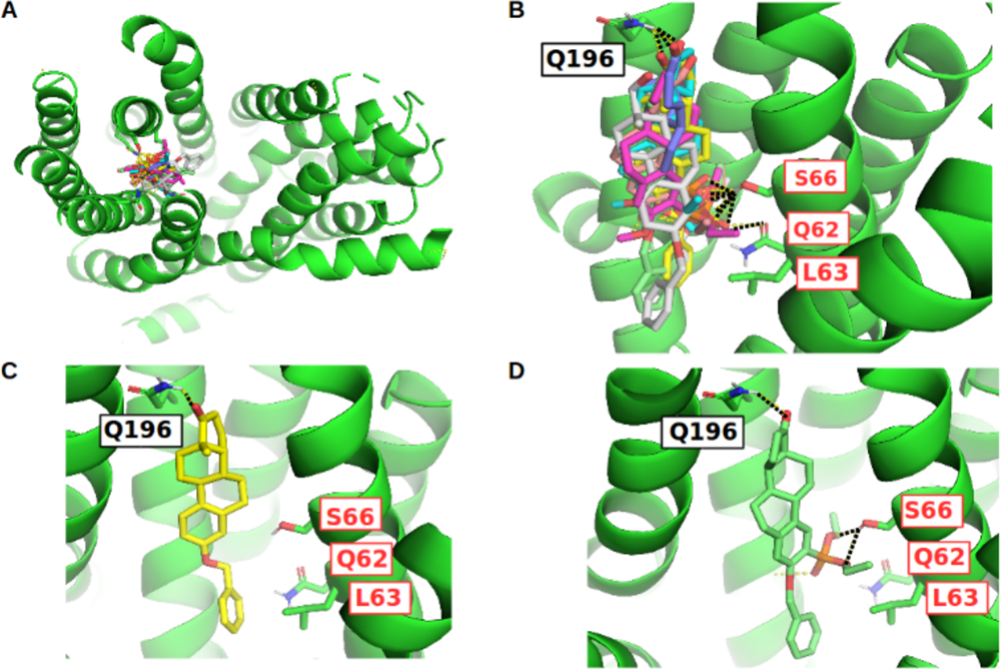
Docking poses of selected 13α-estrone derivatives with a benzyloxy moiety in *R-3*: Top view (A), side view (B), docking pose of compound **3** (C), and compound **11** (D). Hydrogen bonds or other polar contacts are visualized by black dashed lines. The residues labeled in red were validated by mutagenesis studies published in the literature.

Phosphonated 13α-estrone derivatives have previously been reported as strong OATP2B1 inhibitors.^52^ This group of analogous compounds shows a consistent binding pattern with GLN196 (TMH4) and SER66 (TMH1) as main interaction partners (these two residues are also the main interacting residues within the whole group of 13α-estrone derivatives in this study with 41% and 32% frequency, see Table S9). Whereas the carbonyl oxygen at position R-17 position typically forms the H-bond interaction with the GLN196 side chain, H-bond formation between the phosphono group at either R-2 or R-4 position is typically formed with SER66 (or sometimes also with the neighboring GLN62; see Figures 8 and S14). SER66 was reported in literature to be important for transport of endogenous substrates,^72^ and it is interesting that this residue is located at the corresponding position 45 in OATP1B1 and OATP1B3 (which seems to be important for differences in binding in these other two transporters; see previous chapter). Also, GLN62 is an experimentally confirmed functionally important residue in OATP2B1.^72^

The most potent pan-inhibitor out of the six phosphonated 13α-estrone derivatives discussed in this study (compounds **11**–**16**) is carrying the diethyl phosphono group in position R-2 and possesses a benzyloxy moiety at position *R*3̅ (compound **11**; IC_50_ = 0.18 μM). It is at the same time the most potent OATP2B1 inhibitor reported in this study. We observed two possible slightly different binding orientations (occurring with approximately the same frequency) for this compound both possessing the R-17-GLN196 interaction. In the first one (Figures 8D and S15), the diethyl phospho group is oriented toward the inner site of the N-terminal region and forms H-bonds with the side chain of SER66 (TMH1) and GLY203 (TMH4, main chain interaction). The benzyloxy moiety is pointing toward a hydrophobic pocket (enframed by LEU63 at TMH2, ILE206 at TMH4, and LEU230 and PHE231 at TMH5). In the second possible orientation the diethyl phosphono group is interacting with TMH4 exclusively (H-bond formation with the side chain of GLN207), while the benzyl-ether moiety is buried more deeply in the hydrophobic pocket (visualized in Figure S16). In both cases, the hydrophobic interactions of the benzyloxy moiety with its environment seems to be a leading cause for the high affinity observed for this compound since the corresponding 3-hydroxy and 3-methoxy analogs (compound **13** and **15**) are showing an approximately 17-fold decrease in activity (compound poses shown in Figure S16). Also, for 13α-estrone derivatives phosphonated in R-4, we can observe the positive effect on affinity by introducing lipophilicity in position *R*3̅ since compound **12** (benzyloxy moiety in *R*3̅) is the most active of this series (IC_50_ = 0.75 μM), followed by compound **16** (methoxy moiety in *R*3̅, IC_50_ = 2.74 μM), and compound **14** (hydroxyl moiety in *R*3̅, IC_50_ = 10.24 μM).

In general, the R-4-substituted phosphonated 13α-estrone derivatives are a little less active than the equivalent R-2 phosphonated derivatives (especially obvious when comparing the compound pairs **11/12** and **13/14** where a 3- to 4-fold drop in affinity is observed; see Table 1). A possible explanation for this behavior is a smaller tendency to form interactions with SER66 in case of R-4 substitution due to steric reasons. This again highlights the central role of SER66 for OATP2B1 steroid binding. Analogous compounds lacking a diethyl phosphono moiety are still medium to weakly strong inhibitors, showing similar binding poses but lacking the activity determining SER66 interaction (see Figure 8C for compound **3** and Figure S14F for compound **2**).

The majority of the seven halogenated 13α-estrone derivatives reported in this study also act as potent inhibitors of OATP2B1^53^ with IC_50_ ranging from 0.6 to 10.45 μM (Table 1). The position of the halogen substituent seems to play a crucial role here, with R-2 halogenated representatives (compounds **4**, **5**, **7**, and **9**) showing a higher OATP2B1 activity compared to their R-4 counterparts (compounds **6**, **8**, **10**). Comparing the structural analogs, respectively, reveals a 2.5-fold to almost 12-fold difference of the respective binding affinities (Table 1). Interestingly, two distinct interaction sites for 13α-estrone derivatives possessing a halogen at the R-2 (site 1) or the R-4 position (site 2) were identified.

**Figure 9:**
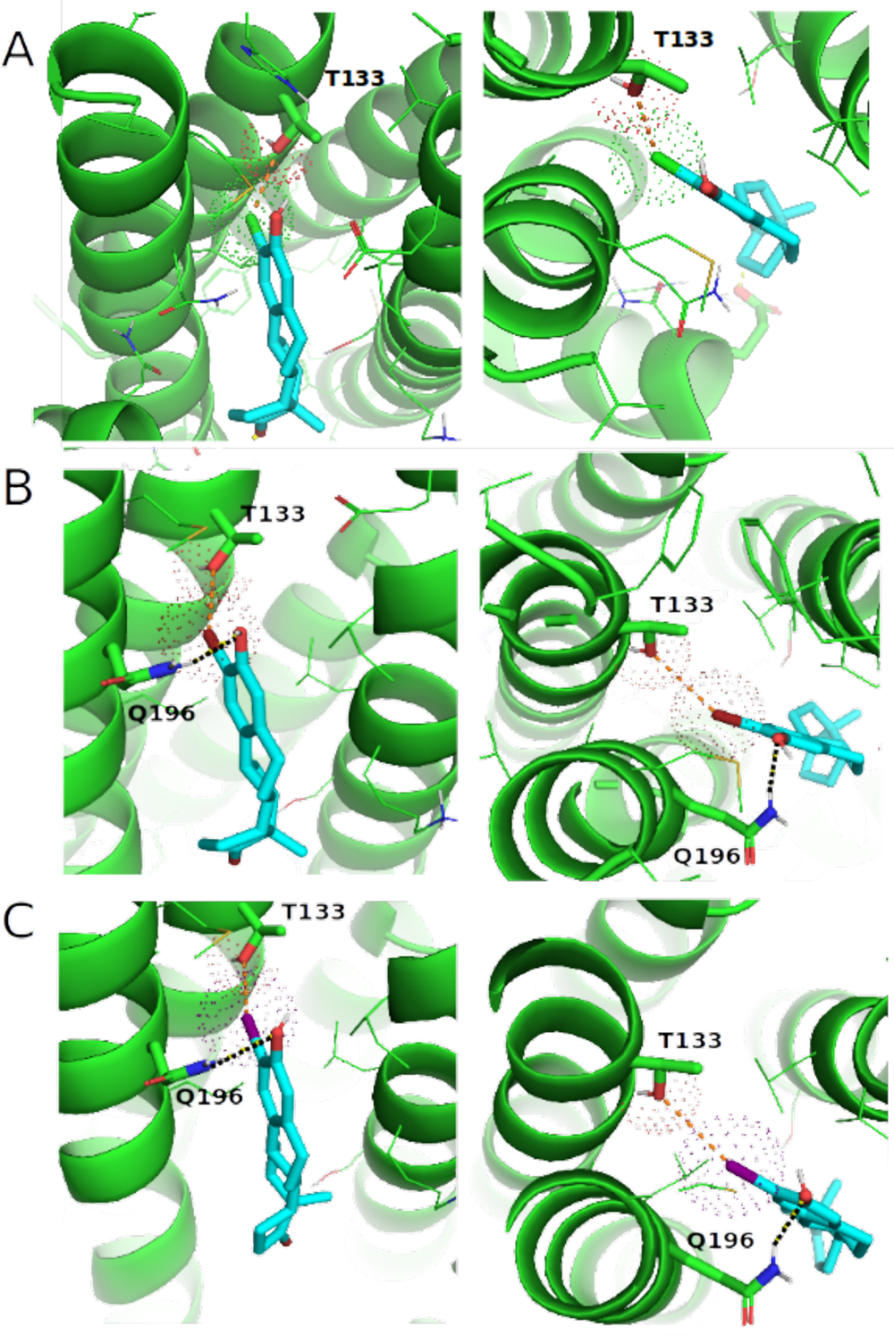
R-2 halogenated 13α-estrone derivatives interacting with site 1: side view (left panel) and top view (right panel). van der Waals radii of the halogen substituents and the carboxyl oxygens of THR133 are visualized as dotted spheres. Halogen bonds are visualized by dashed orange lines. H-bonds are visualized by dashed black lines. (A) Compound **9** (2-chlorine substituent), (B) compound **5** (2-bromine substituent), and (C) compound **7** (2-iodine substituent). Color code: docked compounds = cyan (carbon), red (oxygen), green (chlorine), brown (bromine), purple (iodine); OATP1B1 = blue; OATP1B3 = magenta; and OATP2B1 = green.

Site 1 is located in the upper half of the N-terminal domain (lined by TMH1, TMH2, TMH3, and TMH4) with the *R*3̅ substituent (hydroxyl or methoxy group) of R-2 halogenated steroid analogs pointing toward the EC part of the transporter (Figure 9) and THR133 (TMH3) acts as interaction partner for halogen bond formation. In general, threonine is a known residue capable of forming halogen bonds via its side chain hydroxyl group.^73^ In this study, the likelihood for halogen bond formation has been evaluated on the basis of X---O(THR133) distances and C20-X---O bond angles, where X corresponds to Cl (Figure 9A), Br (Figure 9B), or I (Figure 9C), respectively. Geometric parameters are listed in Table S10. Bond distances are in the range of 3.2–3.9 Å, while bond angles range from 131° to 156°. Comparable geometric parameters were found for available PDB complexes forming halogen bonds, with typical interaction distances ranging from 2.5 to 6.0 Å, while the interaction angles range from 120° to 180°.^74,75^ Therefore, we assume halogen bond formation is the major driver for OATP2B1 interactions with halogenated 13α-estrone derivatives in “site 1”. In addition, the side chain of GLN196 has been found to form a H-bond with the hydroxyl group at position *R*3̅. The 3-methoxy substituent of compound **4** disables the formation of this interaction leading to a 7-fold decrease in affinity for compound **4** compared to its hydroxyl derivative (compound **5**). Since GLN196 is nonconserved (GLY177 in OATP1B1 and OATP1B3), the side chain interaction is also disabled in case of the other two transporters.

In site 2 (observed for steroids halogenated at position R-4), the steroidal core is flipped and the *R*3̅ substituent is pointing toward the IC part (Figure S17). Here, GLN207 and GLN196 are acting as H-bond donor/acceptors, interacting with the hydroxyl group at *R*3̅ and/or the carboxyl oxygen at R-17, respectively. Both interactions are side chain interactions with the amide nitrogen of GLN and are interesting residues to follow up with experiments since also GLN207 is nonconserved (ILE188 in OATP1B1 and OATP1B3). SER66 (TMH1) was found as a potential interaction partner for the R-4 halogenated substituents. However, the bond lengths (ranging from 3.5 to 4.7 Å), as well as bond angles (ranging from 80° to 110°) do not appear geometrically favorable for halogen bond formation (Table S10).

Summarizing the observations from molecular docking of 13α-estrone derivatives into OATP2B1 point to a potential importance of GLN62, SER66, THR133, GLN196, and GLN207 in OATP2B1 transport function. Some of these findings are in accordance with alanine scanning experiments conducted for TMH1 in OATP2B1.^72^ Specifically, GLN62ALA and SER66ALA mutations decreased binding affinity of E-3-S and taurocholate, thus showing an involvement of these two residues in OATP2B1 transport function. The roles of GLN196, THR133, and GLN207 have not yet been confirmed experimentally, but since these residues are nonconserved (compared to the other two hepatic OATPs), they are certainly interesting residues to investigate in future mutational studies.

#### Nonconserved Residues Help Explain OATP2B1 Selectivity

Studying docking poses of 13α-estrone derivatives in all three hepatic OATPs suggested structural determinants for OATP1B/OATP2B1 selectivity of steroid analogs.

For the subset of phosphonated 13α-estrone derivatives, the ones showing activity on OATP2B1 and possessing a benzyloxy or hydroxy group in *R*3̅ (compounds **11**, **12**, and **13**) do also show decent activity on OATP1B1 and OATP1B3 (0.8–5.2 μM). In contrast, OATP2B1-actives with a methoxy group in *R*3̅ (compounds **15** and **16**) are rather inactive on the other two transporters (9.5–32.4 μM).

Compounds **11** and **12** which are possessing a benzyloxy group at position *R*3̅ are adopting similar vertical binding modes in OATP1B1/OATP1B3 at the TMH1/TMH2 interface as seen for OATP2B1 complexes. However, an interaction of the R-17 carbonyl group (with GLN196) is disabled since in OATP1B1/OATP1B3 GLN196 is exchanged with GLY177. The second frequently observed side chain interaction in OATP2B1, formed by SER66 with the diethyl phosphono group, is also disabled by exchange of this residue to ALA45 (OATP1B1) and GLY45 (OATP1B3). Instead, the R-17 carbonyl group interacts with other residues from TMH4 (e.g., ASN178 in OATP1B1). In addition, hydrophobic contacts via its benzoyloxy side chain are providing an explanation why these compounds are also highly active in OATP1B1 and OATP1B3 (Figure S18).

Compound **13** seems to exhibit its main interaction in OATP1B1 and OATP1B3 via H-bond formation of its 3-OH group with GLU185 leading to moderate activity on these transporters despite missing hydrophobic interactions. The 3-methoxy derivatives (compounds **15** and **16**) are rather inactive on OATP1B1/OATP1B3 probably due to a lack of all main interactions observed for OATP2B1 binding (Figure S16) with the additional flaw of not being able to form strong hydrophobic contacts or direct H-bonds via its *R*3̅ substituent.

Halogenated derivatives show a tendency to be more active on or even selective (compounds **4**, **5**, **7**, and **9**) for OATP2B1. Interestingly, these selective compounds are all halogenated in position R-2, whereas the R-4 halogenated compounds (compound **6** and **8**) tend to be also active on OATP1B1 and OATP1B3 although with weak to borderline inhibitory activity.

In OATP2B1, the formation of halogen bonds for R-4 halogenated 13α-estrone derivatives with SER66 seems less geometrically favorable than for R-2 halogenated compounds. Thus, loss of this interaction when binding to OATP1B1 or OATP1B3 (through replacement with A45 or G45) is not expected to lead to a drop in affinity. However, in OATP1B1 and OATP1B3, a certain preference of R-4 halogenated substituents to bind to the central cavity rather than in the N-terminal domain with only a few distinct interactions have been observed (e.g., with LYS49, ASN77), leading to their quite weak activities.

For 13α-estrone derivatives halogenated in R-2 (compounds **4**, **5**, **7**, and **9**) reasons why OATP2B1 selectivity occurs could be manifold. Certainly, the missing halogen bonding partner (THR133; ALA112 and SER112 in OATP1B1 and OATP1B3, respectively) as well as the missing GLN196 (GLY177 in OATP1B1 and OATP1B3) for side chain interactions with the 3-OH group play a big role.

It appears interesting to compare the identified halogen binding site in OATP2B1 (site 1) to the putative sites in OATP1B1/OATP1B3 (see Figure S19). Mapping an electrostatic surface onto the transporter structure reveals that the N-terminal binding pocket is highly positively charged in OATP1B1/OATP1B3, compared to the OATP2B1. The increased electrostatic surface in OATP1B1 and OATP1B3 is caused by the presence of positively charged residues (such as LYS41 and LYS49), which are replaced by uncharged residues in OATP2B1. As halogens prefer to bind to a hydrophobic environment^76^ the preference for OATP2B1 becomes evident.

Overall, the conclusions drawn from these observations suggest that selectivity of hepatic OATPs is controlled by a limited number of nonconserved residues at the TMH1/TMH2 interface, as well as in TMH4, which can affect specific interactions as well as pocket size.

## Summary and Conclusions

Molecular modeling of OATP-ligand interactions remains challenging because of the lack of detailed knowledge about the protein structure. Here, we present an integrative computational approach, involving a systematic exploration of available structures with MFS-fold by normal-mode analysis, construction of multiple OATP models based on alternate conformations of selected templates, prioritization of the models on the basis of enrichment docking, and an in-depth analysis of molecular interactions for steroid analogs.

Signature dynamics of MFS proteins shows conserved fluctuations of specific protein subdomains. Among others, an increased flexibility at the TMH1/TMH2 interface was found to be an important determinant which contributes to the intrinsic dynamics of MFS proteins. These findings suggest functional importance of the TMH1/TMH2 interface. Therefore, the selected structural template (here: FucP transporter) was sampled along these modes in such a way that the final conformational ensemble covers movement of TMH1 and TMH2 helices (and TMH7 and TMH11 to a lesser extent). The calculated network models of the template were subsequently used to build OATP structural models in different conformations. Enrichment docking of a large data set retrieved from the open domain (spiked with decoys from DUD-E database) was employed to validate the structural models on the basis of their ability to enrich known actives. Top prioritized models for OATP1B1, OATP1B3, and OATP2B1, are showing an out-of-plane shift of their TMH1/TMH2 helices. Structural models exhibit a different shape of the N-terminal binding site compared to the ones generated on the basis of the initial template structure. Interestingly, by retrospective redocking of steroid analogs into the models based on the initial template structure, the established binding modes for steroids could not be fully reproduced leading to distinct binding modes which did only partially correspond to experimentally proven ligand-protein interactions. This finding indirectly proves our strategy to be valid and useful.

Also, earlier mutational experiments for OATP1B1 have shown an involvement of residues at TMH1 (LYS41, GLY/ALA45, LYS49)^71^ and TMH2 (ASP70, PHE73, GLU74, and GLY76)^77^ in the transport of natural substrates (mostly E-3-S or taurocholate). Similarly, alanine scanning of TMH1 on OATP2B1 helped identify key residues (VAL52, HIS55, GLN59, ALA61, GLN62, SER66, and LEU69) implicated in the transport of E-3-S and taurocholate.^72^ Thus, interactions of ligands with the TMH1/TMH2 interface seem to be of high relevance, as also shown by the docking results for steroid analogs in this study.

Docked steroids generally show orientational versatility in the binding sites. Specifically, the R-3 and R-17 substituents are capable of forming most of the key interactions.

Cluster analysis reveals two distinct sites for OATP1B1/OATP1B3—one site in the central cavity and one N-terminal binding site. Experimental data showed that several steroids (such as E-3-S) exhibit biphasic kinetics, which led to the identification of low- and high-affinity binding sites. Specifically, Li et al.^27^ have shown that the alanine mutants of ASP70 in OATP1B1 affect both low- and high-affinity components, while PHE73, GLU74, and GLY76 only affects a single site. ASP70 is located in the upper part of TMH2, thus being partially involved in both the N-terminal binding site and the central cavity. The other mutated residues are largely oriented toward the central cavity. In another study on OATP1B1, LYS41, and LYS49 (both located at THM1) showed impaired *K*_m_ values at the high- and low-affinity binding site of E-3-S, respectively. Moreover, LYS41ALA mutation led to similar *K*_m_ values compared to the wild-type transporter for the low-affinity site of E-3-S, thus suggesting that LYS41 is only implicated in a single (high-affinity) binding site. In contrast, LYS49ALA mutation affected the low affinity component of E-3-S. By comparing these findings to our OATP structural models, we reveal that LYS41 is buried in the N-terminal binding region, while LYS49 is exposed more toward the central cavity of the transporter. Overall, the experimental data reported in literature suggest that the N-terminal region might correspond to the high-affinity binding site for steroids, while the central region represents the low-affinity binding site. However, to fully assess which one of the two regions identified in this study might represent the high- or low-affinity site, free energy calculations have to be undertaken in future studies.

For selecting the best model for steroid docking into OATP2B1, a data set largely lacking steroidal structures from the open domain was used for the enrichment docking procedure. By this procedure an unbiased structural model, not trained to recognize specifically steroids, could be obtained. The in-house data set with 16 13α-estrone derivatives served as a validation set for all three transporters but was especially effective in case of OATP2B1, since 14 compounds of this data set are showing strong (0.18 μM) to medium inhibitory activity (8.39 μM) toward this target (with the exception of 13α-estrone and compound **10**). In contrast to the other two transporters, OATP2B1 shows a single, N-terminal binding site, for interactions with steroid analogs. However, we have to emphasize that the conclusions drawn for OATP2B1 might be affected by the limited structural diversity of the OATP2B1 data set. By comparing interactions adapted by OATP1B1 and OATP1B3 in the N-terminal binding site, differences in ligand accessibility appear to be the main cause for variations in ligand binding. This is partly affected by differently formed salt bridges in the cavity, as well as by the replacement of alanine (OATP1B1) at positions 45 and 216 to glycine (OATP1B3), which leads to disparities in pocket geometry. In OATP2B1, a single N-terminal binding site at the TMH1/TMH2 interface accommodates 13α-estrone derivatives with OATP2B1 activity. A distinct halogen binding site with the likelihood of halogen bond formation in the upper part of the N-terminal half was identified (at THM3). The corresponding halogen binding site was not found in OATP1B1 and OATP1B3, likely due to replacement of THR133 (in OATP2B1) to ALA112 (in OATP1B1) and SER112 (in OATP1B3), which might explain selectivity or stronger binding affinity toward OATP2B1 for halogenated compounds Moreover, OATP2B1-specific binding of halogenated 13α-estrone derivatives likely happens because of the different compositions of the electrostatic surface in OATP1B1/1B3 (positively charged) versus OATP2B1 (hydrophobic). As a follow up study, we plan to perform quantum mechanics optimization of the binding modes possessing halogen bonding.

Interestingly, a single residue at TMH1 (residue number 45 in OATP1B1/1B3, 66 in OATP2B1) seems to play a role as OATP selective switch given its role as a steric hindrance and ability to adopt hydrogen bonds. In this Article, chemical specificity was exemplified, for example, for phosphonated 13α-estrone derivatives by their ability to form a hydrogen bond with SER66 in OATP2B1 (but not with ALA45 or GLY45 in OATP1B1/1B3). Steric effects are potentially the reason for digoxin selectivity toward OATP1B3, where the size of the binding site is crucial for accommodating its bulky substituent at position R-3 of the steroidal core. As the N-terminal binding site in OATP1B3 possesses the highest volume—given the loss of the side chain at position 45 and 216, respectively (both GLY)—the OATP1B3-selective binding of digoxin can be explained. The role of residue number 45 for OATP1B1/1B3 selectivity was previously confirmed by mutational studies replacing GLY45 (OATP1B3) to ALA45 (OATP1B1) and vice versa.^71^ Herein, we also found an indication that binding of steroids to SER66 in OATP2B1 could drive activity toward this transporter (as also shown in OATP2B1 mutational studies),^72^ and that ALA45/GLY45/SER66 might be responsible for driving selectivity of steroid analogs across the three hepatic OATPs.

The TMH1/TMH2 interface has previously been identified as an essential substrate binding cavity for different types of MFS proteins, including the POT family of oligopeptide transporters.^78^ In future studies, different transporters with MFS fold should be explored to investigate whether ligand recognition at the N-terminal domain of MFS transporter represents a consistent pattern.

Apart from the interesting interactions happening at TMH1/TMH2, we also observed a crucial involvement of nonconserved residues at TMH3 and TMH4, especially THR133 in OATP2B1 (ALA112/SER112 in OATP1B1/OATP1B3), GLN196 (GLY177), and GLN207 (ILE188). These are interesting residues to follow up on with mutational studies since our computational experiments are pointing toward their important roles in conferring OATP2B1 selectivity.

With this study, we ultimately contributed to the knowledge about structural determinants of steroids’ binding to hepatic OATPs, using a rigorous computational protocol for generating structural models, followed by the comparative analysis of important ligand interactions at the identified binding sites. Insights about interactions of steroid analogs with OATP1B1, OATP1B3, and OATP2B1 are contributing to the knowledge of compound requirements for the design of new chemical probes, which can further elucidate the physiological role of these emerging transporters.

## Supporting Information Available

Residues known to be implicated in transport function or transporter specificity; five top ranked templates predicted for OATP1B1, OATP1B3, and OATP2B1; table listing amino acid residues spanning the different transmembrane regions for the three hepatic OATPs; AUC values for top five models per transporter; crystal structures of MFS transporters identified on the basis of signature dynamics investigations; cluster analysis for docking of steroidal compounds into OATP1B1; cluster analysis for docking of steroidal compounds into OATP1B3; cluster analysis for docking of steroidal compounds into OATP2B1; frequency of interactions formed by steroid analogs with OATP1B1, OATP1B3, and OATP2B1; geometric parameters for the halogen-bond formation; counts of protein–ligand interactions per R-group position; five lowest frequency modes calculated for the selected template; covariance matrices showing the sequence (Hamming distance), secondary structure (RMSD), and dynamic similarity between retrieved MFS transporters; enrichment docking into OATP1B1/OATP1B3/OATP2B1 structural models; comparison of the top prioritized structural models for OATP1B1, OATP1B3, and OATP2B1; protein–ligand interactions fingerprints for top three enriched OATP1B1 clusters; protein–ligand interactions fingerprints for top two enriched OATP1B3 clusters; protein–ligand interactions fingerprints for the top enriched OATP2B1 cluster; key amino acid residues in OATP1B1/OATP1B3/OATP2B1 predicted to interact with steroid analogs; identified intramolecular salt bridges in hepatic OATPs; comparison of interactions of steroids in the N-terminal binding site of OATP1B1 and OATP1B3; binding mode of E-3-S in OATP1B1 suggesting OATP1B1 selectivity; binding modes of selected 13α-estrone derivatives in OATP2B1; docking pose of compound 11 visualized with van der Waals radii of the ligand and its surrounding environment; binding modes of R-2 phosphonated 13α-estrone derivatives in OATP2B1; R-4 halogenated 13α-estrone derivatives binding in close proximity to SER66 in OATP2B1; binding of compound 11 and compound 12 in OATP1B1 and OATP1B3 transporters; putative halogen binding site in OATP1B1 and OATP1B3 compared to the docked pose in OATP2B1; and structural models for OATP1B1, OATP1B3, and OATP2B1 (PDF)

## Notes

Supplementary files containing an alignment file used for the comparative modeling of OATP1B1 (OATP1B1.ali), an alignment file used for the comparative modeling of OATP1B3 (OATP1B3.ali), an alignment file used for the comparative modeling of OATP2B1 (OATP2B1.ali), a CSV file listing steroid analogs gathered from the open data sources, and a python script for retrieving the maximum common substructure from 3D ligand structures are also available from GitHub: https://github.com/AlzbetaTuerkova/EnsembleDocking.

## Abbreviations

ANM: anisotropic network model
CCK-8: cholecystokinin-8
DHEA: dehydroepiandrosterone
DSSP: define secondary structure of proteins
EC: extracellular
E-3-S: estrone-3-sulfate
FucP: fucose transporter
GNM: Gaussian network model
IC: intracellular
MFS: major facilitator superfamily
NMA: normal mode analysis
OATP: organic anion transporting polypeptide
PLIF: protein–ligand interaction fingerprint
SLC: solute carrier
TMH: transmembrane helix
