# Supplementary material for: Data-Driven Ensemble Docking to Map Molecular Interactions of Steroid Analogs with Hepatic Organic Anion Transporting Polypeptides
Source: J Chem Inf Model. 2021 Jun 9;61(6):3109–27. doi: 10.1021/acs.jcim.1c00362 (PMC8243326; doi:10.1021/acs.jcim.1c00362)
Supplement: Supplementary file 1 — ci1c00362_si_001.pdf [file ci1c00362_si_001.pdf]

Supporting Information

Data-driven Ensemble Docking to Map Molecular Interactions of Steroid Analogs with Hepatic Organic Anion Transporting Polypeptides

Alzbeta Tuerkova,[1,†] *Orsolya Ungvári,*[2] *Réka Laczkó-Rigó,*[2] *Erzsébet Mernyák,*[3] *Gergely Szakács,*[2,4] *Csilla Özvegy-Laczka,*[2] *Barbara Zdrazil*[1,*]

[1] University of Vienna, Department of Pharmaceutical Sciences, Division of Pharmaceutical Chemistry, Althanstraße 14, A-1090 Vienna, Austria

[2] Drug resistance Research Group, Institute of Enzymology, RCNS, Eötvös Loránd Research Network, H-1117, Budapest, Magyar tudósok krt. 2, Hungary

[3] Department of Organic Chemistry, University of Szeged, Dóm tér 8, H-6720 Szeged, Hungary

[4] Department of Medicine I, Institute of Cancer Research, Comprehensive Cancer Center, Medical University of Vienna, Vienna, Austria

*Correspondence: barbara.zdrazil@univie.ac.at

Table S1. Residues known to be implicated in transport function or transporter specificity (highlighted in color): Transmembrane helix number, amino acid, probe substrate, and reference to the primary source (PubMed ID or DOI) are indicated. Residues which were observed as interacting partners in docking poses of steroidal compounds in this study are highlighted by a red font. TM…..Transmembrane Helix; E-3-S…..estrone-3-sulfate.

TM	Amino acid	Probe substrate	OATP1B1	OATP1B3	OATP2B1
1	-----/-----/V52	E-3-S, taurocholic acid			29871943
1	L34/L34/H55	E-3-S, taurocholic acid			29871943
1	S37/S37/L58	E-3-S, taurocholic acid			29871943
1	F38/Y38/Q59	E-3-S, taurocholic acid			29871943
1	A40/A40/A61	E-3-S, taurocholic acid			29871943
1	K41/K41/Q62	E-3-S	31254566	20100277	29871943
1	A45/G45/S66	cholecystokinin-8, E-3-S, taurocholic acid, epigallocatechin gallate	22352740	22352740 31353905	29871943
1	I46/I46/G67	E-3-S	31254566		
1	M48/M48/L69	E-3-S, taurocholic acid			29871943
1	K49/K49/K70	E-3-S	31254566		
1	R57/R57/K78	estradiol-17β-glucuronide, E-3-S, bromo-sulfophthalein	20821001		
2	D70/D70/A91	E-3-S	22574206		

2	F73/F73/N94	E-3-S, estradiol 17beta-D-glucuronide	22574206, 11477075		
2	E74/E74/E95	E-3-S	22574206		
2	G76/G76/G97	E-3-S	22574206		
4	V174/V174/M192	E-3-S, SN-38, pravastatin, estradiol-17beta-glucuronide	15608127		
5	R181/R181/L199	estradiol-17β-glucuronide	https://doi.org/10.1096/fasebj.21.5.A196-d		
6	W258/W258/W276	taurocholic acid, E-3-S	23858103		
6	W259/W259/W277	taurocholic acid, E-3-S	23858103		
7	I353/I353/A388	E-3-S, estradiol 17beta-D-glucuronide	11477075		
7	K361/K361/K386	E-3-S, estradiol-17β-glucuronide, bromo-sulfophthalein	20821001	21642393	
10	F537/Y537/F564	cholecystokinin-8		18690707	
10	L545/S545/S572	cholecystokinin-8, E-3-S	22352740, 19760661	22352740,18690707	
10	F546/L546/A573	E-3-S	19760661		
10	L550/T550/L577	E-3-S, cholecystokinin-8	19760661	18690707	
10	G552/G552/H579	E-3-S, pravastatin, rosuvastatin, sulfasalazine, naringin, progesterone			https://doi.org/10.1039/C6MD00235H

10	S554/T554/P581	E-3-S	19760661		
10	H555/F555/S582	epigallocatechin gallate		31353905	
11	R580/R580/R607	E-3-S, estradiol-17β-glucuronide, bromosulfophthalein	20821001, https://doi.org/10.1096/fasebj.21.5.A196-d	20100277	
12	F591/F591/H618	E-3-S, pravastatin, rosuvastatin, sulfasalazine, naringin, progesterone			https://doi.org/10.1039/C6MD00235H

Table S2. Five top ranked templates predicted for (A) OATP1B1, (B) OATP1B3, (C) OATP2B1.

(A)

Net Score	p-value	Alignment length	Template length	Target length	PDB ID	Resolution [Å]
82.116	3e-07	402	409	691	6e9n	2.92
79.538	5e-07	414	453	691	3wdo	3.15
78.253	6e-07	426	465	691	6e8j	3.09
**75.504**	**1e-06**	**430**	**434**	**691**	**3o7q**	**3.14**
73.959	2e-06	404	414	691	4av3	2.60

(B)

Net Score	p-value	Alignment length	Template length	Target length	PDB ID	Resolution [Å]
74.704	1e-06	400	409	702	6e9n	2.92
**74.551**	**2e-06**	**403**	**414**	**702**	**3o7q**	**3.14**
70.557	4e-06	428	434	702	1pw4	3.30
69.415	5e-06	202.0	404	702	6e8j	3.09
69.372	5e-06	236.0	432	702	1wa5	2.00

(C)

**Net Score**	**p-value**	**Alignment length**	**Template length**	**Target length**	**PDB ID**	**Resolution [Å]**
**93.148**	**2e-08**	**401**	**414**	**709**	**3o7q**	**3.14**
86.484	9e-08	419	453	709	3wdo	3.15
84.431	2e-07	434	465	709	6e8j	3.09
83.100	2e-07	399	409	709	6e9n	2.92
83.039	2e-07	433	434	709	1pw4	3.30

Table S3. Table listing amino acid residues spanning the different transmembrane regions for the three hepatic OATPs.

TMH#	amino acid sequence
1	OATP1B1: **28**K----MFLAALSLSFIAKTLGAIIMKSSIIHIERR**58**
	OATP1B3: **28**K----MFLAALSFSYIAKALGGIIMKISITQIERR**58**
	OATP2B1: **49**KLFVL----CHSLLQLAQLMISGYLKSSISTVEKR**79**
	Cons: ......*....*........*.**...*.*
2	OATP1B1: **64**SLVGFIDGSFEIGNLLVIVFVSYFGSKLHR**93**
	OATP1B3: **64**SLAGLIDGSFEIGNLLVIVFVSYFGSKLHR**93**
	OATP2B1: **85**QTSGLLASFNEVGNTALIVFVSYFGSRVHR**114**
	Cons: ...*......*.**...*********..**
3	OATP1B1: **94**PKLIGIGCFIMGIGGVLTALPHFF**117**
	OATP1B3: **94**PKLIGIGCLLMGTGSILTSLPHFF**117**
	OATP2B1:**115**PRMIGYGAILVALAGLLMTLPHFI**138**
	Cons: *..**.*.........*..****.
4	OATP1B1:**171**WIYVFMGNMLRGIGETPIVPLGLSYI**196**
	OATP1B3:**171**WIYVFMGNMLRGIGETPIVPLGISYI**196**
	OATP2B1:**189**VGIMFVAQTLLGVGGVPIQPFGISYI**214**
	Cons: ....*....*.*.*..**.*.*.***
5	OATP1B1:**204**HSSLYLGILNAIAMIGPIIGFTLGSLF**230**
	OATP1B3:**204**HSSLYLGSLNAIGMIGPVIGFALGSLF**230**
	OATP2B1:**222**NSPLYLGILFAVTMMGPGLAFGLGSLM**248**
	Cons: .*.****.*.*..*.**...*.****.
6	OATP1B1: **258**WWLNFLVSGLFSIISSIPFFF**278**
	OATP1B3: **258**WWLGFLVSGLFSIISSIPFFF**278**
	OATP2B1: **276**WWLGFLIAAGAVALAAIPYFF**296**
	Cons: ***.**.........**.**
7	OATP1B1:**324**GFFQSFKSILTNPLYVMFVLLTLLQVSSYIGAFTYVFKYVEQQ**366**
	OATP1B3:**324**GFFQSLKSILTNPLYVIFLLLTLLQVSSFIGSFTYVFKYMEQQ**366**
	OATP2B1:**359**VFPRVLLQTLRHPIFLLVVLSQVCLSSMAAGMAIFLPKFLERQ**401**
	Cons: .*.......*..*......*......*...*......*..*.*
8	OATP1B1: **373**KANILLGVITIPIFASGMFLGGYIIK**398**
	OATP1B3: **373**HANFLLGIITIPTVATGMFLGGFIIK**398**
	OATP2B1: **408**YANLLIGCLSFPSVIVGIVVGGVLVK**433**
	Cons: .**.*.*....*....*...**...*
9	OATP1B1: **405**VGIAKFSCFTAVMSLSFYLLY**425**
	OATP1B3: **405**VGIAKFSFLTSMISFLFQLLY**425**
	OATP2B1: **440**VGCGALCLLGMLLCLFFSLPL**460**
	Cons: **..............*.*..
10	OATP1B1: **533**FFYFFVAIQVLNLFFSALGGTSHVML**558**
	OATP1B3: **533**FFFIYVAIQVINSLFSATGGTTFILL**558**
	OATP2B1: **560**FVVPFLLLVSLGSALACLTHTPSFML**585**
	Cons: *..................*....*

11	OATP1B1:	**570**LALGFHSMVIRALGGILAPIYFGALIDT**597**
	OATP1B3:	**570**LAMGFQSMVIRTLGGILAPIYFGALIDK**597**
	OATP2B1:	**597**LAVGIQFMFLRILAWMPSPVIHGSAIDT**624**
	Cons:	**.*...*..*.*.....*...*..**.
12	OATP1B1:	**598**TFSRVYLGLSSMLR------VSSLVL**639**
	OATP1B3:	**598**TFGRVYLGLSIALRFPALVL------**639**
	OATP2B1:	**625**TLRNRFIGLQFFFK------TGSVIC**665**
	Conf:	*......**..... ....

Table S4. AUC values for top five models per transporter.

Transporter	AUC
OATP1B1	0.830
OATP1B1	0.821
OATP1B1	0.810
OATP1B1	0.810
OATP1B1	0.810
OATP1B3	0.940
OATP1B3	0.930
OATP1B3	0.930
OATP1B3	0.917
OATP1B3	0.909
OATP2B1	0.694
OATP2B1	0.680
OATP2B1	0.672
OATP2B1	0.672
OATP2B1	0.661

Table S5. Crystal structures of MFS transporters identified on the basis of signature dynamics investigations: PDB ID and the corresponding title are listed here. If a certain PDB structure was identified as a suitable template for one of the hepatic OATPs, the prediction score from pGenThreader algorithm is listed in this table. NA…..not available.

PDB ID	Title	Score OATP1B1	Score OATP1B3	Score OATP2B1
3o7q	Crystal structure of a Major Facilitator Superfamily (MFS) transporter, FucP, in the outward conformation	**75.504**	**74.551**	**93.148**
4m64	3S crystal structure of na+/melibiose symporter of salmonella typhimurium	**79.538**	NA	NA
3wdo	Structure of e. coli YATJ transporter	**79.538**	**69.415**	**86.484**
4gby	The structure of the MFS (Major Facilitator Superfamily) proton:xylose symporter XYLl bound to d-xylose	NA		NA
4gbz	The structure of the MFS (Major Facilitator Superfamily) proton:xylose symporter XYLe bound to d-glucose	NA	NA	NA
4gc0	The structure of the MFS (major facilitator superfamily) proton:xylose symporter XYLe bound to 6-bromo-6-deoxy-d-glucose	**57.105**	NA	NA
4zwc	Crystal structure of maltose-bound human GLUT3 in the outward-open conformation at 2.6 Angstrom	NA	NA	NA
4zw9	Crystal structure of human GLUT3 bound to d-glucose in the outward-occluded conformation at 1.5 Angstrom	NA	NA	NA
5c65	Structure of the human glucose transporter GLUT3 / slc2a3	NA	NA	NA
4jre	Crystal structure of nitrate/nitrite exchanger nark with nitrite bound	NA	NA	NA
4zwb	Crystal structure of maltose-bound human GLUT3 in the outward-occluded conformation at 2.4 Angstrom	NA	NA	NA
4oaa	Crystal structure of e. coli lactose permease g46w, g262w bound to sugar	NA	NA	NA

4zyr	Crystal structure of e. coli lactose permease g46w/g262w bound to p-nitrophenyl alpha-d-galactopyranoside (alpha-npg)	NA	NA	NA
4gxb	Structure of the snx17 atypical ferm domain bound to the npxy motif of p-selectin	NA	NA	NA
3o7p	Crystal structure of the e.coli fucose:proton symporter, FucP (n162a)	NA	NA	NA
5gxb	Crystal structure of a LacY/nanobody complex	NA	NA	NA

Table S6. Cluster analysis for docking of steroidal compounds into OATP1B1 (total number of actives: 20): Selected clusters are highlighted in bold.

ID	% unique compounds	# poses/compound in a cluster			
		Mean	Median	Min	Max
6	85	2	1	1	5
5	65	1.692	1	1	3
2	55	1.727	1	1	4
3	50	1.3	1	1	3
7	45	1.444	1	1	3
9	42	1	1	1	1
1	35	1.714	1	1	4
4	35	1.714	1	1	4
13	30	1	1	1	1
15	25	1.4	1	1	2
14	20	1	1	1	1
8	15	1.333	1	1	2
10	15	1.333	1	1	2
11	15	1	1	1	1
12	15	1.333	1	1	2

Table S7. Cluster analysis for docking of steroidal compounds into OATP1B3 (total number of actives: 12): Selected clusters are highlighted in bold.

**ID**	**% unique compounds**	**# poses/compound in a cluster**			
		Mean	Median	Min	Max
1	100	4.333	4	1	7
2	42	1.6	2	1	2
6	42	1.6	1	1	3
9	42	1	1	1	1
4	33	1.4	1	1	3
7	25	1.333	1	1	2
3	16	1.5	1.5	1	2
5	16	2.5	2.5	2	3
8	16	2	2	1	3

Table S8. Cluster analysis for docking of steroidal compounds into OATP2B1 (total number of actives: 16): The selected cluster is highlighted in bold.

ID	% unique compounds	# poses/compound in a cluster			
		Mean	Median	Min	Max
1	94	5.066	5	3	8
9	60	1.666	2	1	2
5	40	1	1	1	1
2	33	1	1	1	1
4	33	1.8	2	1	3
6	26	1.25	1	1	2
8	26	1.25	1	1	2
3	20	1.333	1	1	2
7	20	1.666	2	1	2

Table S9. Frequency of interactions formed by steroid analogs with OATP1B1, OATP1B3, and OATP2B1: % of all possible interactions, % of all poses (indicated in brackets) is listed in the table. TMH numbers are annotated in the left column.

TMH	OATP1B1		OATP1B3		OATP2B1	
1	**PHE38**	0% (0% poses)	**TYR38**	4%(4% poses)	**GLN59**	0%(0% poses)
1	**LYS41**	0% (0% poses)	**LYS41**	13%(8% poses)	**GLN62**	15%(10% poses)
1	**THR42**	0% (0% poses)	**ALA42**	8%(6% poses)	**LEU63**	5%(5% poses)
1	**ALA45**	0% (0% poses)	**GLY45**	6%(6% poses)	**SER66**	32%(27% poses)
1	**LYS49**	5%(5% poses)	**LYS49**	17%(17% poses)	**LYS70**	0%(0% poses)
2	**ASP70**	3%(3% poses)	**ASP70**	0% (0% poses)	**ALA91**	0%(0% poses)
2	**PHE73**	5%(5% poses)	**PHE73**	0% (0% poses)	**ASN94**	2%(2% poses)
2	**GLU74**	5%(5% poses)	**GLU74**	2%(2% poses)	**GLU95**	7%(7% poses)
2	**ASN77**	25%(13% poses)	**ASN77**	0%(0% poses)	**ASN98**	0%(0% poses)
3	**GLY105**	0% (0% poses)	**GLY105**	2%(2% poses)	**ALA126**	0%(0% poses)
3	**ALA112**	0% (0% poses)	**SER112**	0%(0% poses)	**THR133**	12%(12% poses)
4	**TYR173**	0% (0% poses)	**TYR173**	0%(0% poses)	**ILE191**	2%(2% poses)
4	**ASN178**	10%(10% poses)	**ASN178**	0%(0% poses)	**GLN196**	41%(29% poses)
4	**ARG181**	18%(10% poses)	**ARG181**	0%(0% poses)	**LEU199**	0%(0% poses)
4	**GLY182**	7%(7% poses)	**GLY182**	0%(0% poses)	**GLY200**	0%(0% poses)
4	**GLU185**	8%(8% poses)	**GLU185**	17%(19% poses)	**GLY203**	12%(10% poses)
4	**VAL189**	8%(8% poses)	**VAL189**	13%(10% poses)	**GLN207**	7%(5% poses)
5	**ASN213**	0% (0% poses)	**ASN213**	13%(6% poses)	**PHE231**	0%(0% poses)
5	**ALA216**	2%(2% poses)	**GLY216**	6%(6% poses)	**MET234**	0%(0% poses)
5	**MET217**	2%(2% poses)	**MET217**	2%(2% poses)	**MET235**	0%(0% poses)
5	**GLY219**	0% (0% poses)	**GLY219**	0%(0% poses)	**GLY237**	2%(2% poses)
7	**THR345**	16% (16% poses)	**THR345**	0%(0% poses)	**GLN380**	0%(0% poses)
7	**GLN348**	5%(5% poses)	**GLN348**	6%(4% poses)	**LEU383**	0%(0% poses)
7	**VAL349**	3%(3% poses)	**VAL349**	0%(0% poses)	**SER384**	0%(0% poses)
7	**TYR352**	7%(7% poses)	**PHE352**	0%(0% poses)	**ALA387**	0%(0% poses)
10	**GLY552**	12%(12% poses)	**GLY552**	0%(0% poses)	**HIS579**	0%(0% poses)
11	**SER576**	7%(7% poses)	**SER576**	0%(0% poses)	**PHE603**	0%(0% poses)
11	**MET577**	5%(5% poses)	**MET577**	10%(8% poses)	**MET604**	0%(0% poses)
11	**ARG580**	0% (0% poses)	**ARG580**	15%(13% poses)	**ARG607**	0%(0% poses)

Table S10. Geometric parameters for the halogen-bond formation. Values were measured in PyMOL.

Compound ID	Interact. residue	Distance [A]	Angle [atom selection]	Angle [degree]	Bioactivity value [µM]
Compound 9	THR133	3.2	C20-Cl1---OG1(T133)	131	0.90
Compound 7	THR133	3.9	C20-I1---OG1(T133)	156	0.60
Compound 5	THR133	3.8	C20-Br1---OG1(T133)	155	1.19
Compound 4	THR133	3.7	C2-Br3---OG1(T133)	154	8.39
Compound 10	SER66	3.3	C19-Cl1---OG(S66)	110	10.50
Compound 6	SER66	4.6	C19-Br1---OG(S66)	80	2.97
Compound 8	SER66	4.6	C19-I1---OG(S66)	80	3.58

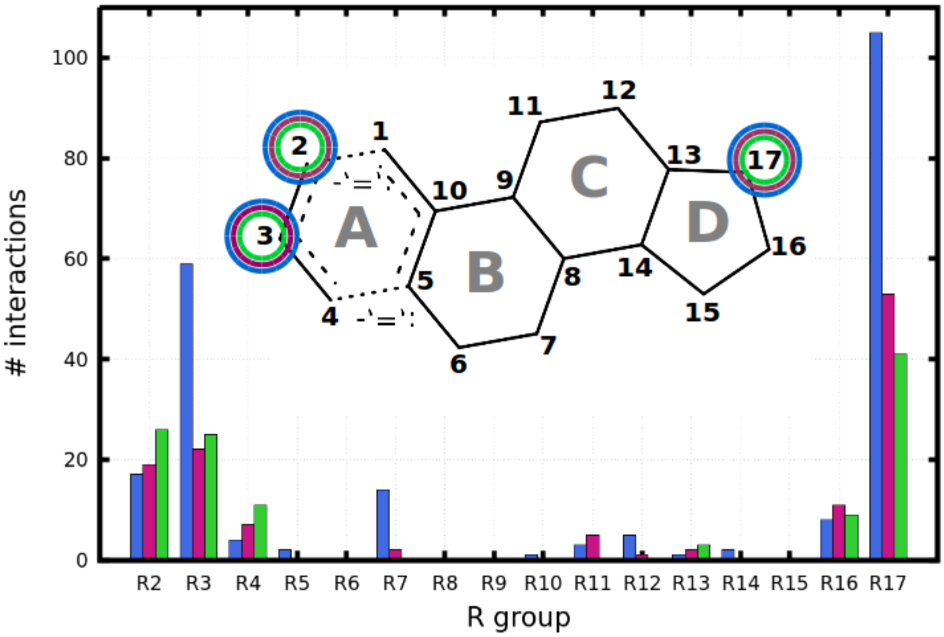

Figure S1. Counts of protein-ligand interactions per R-group position (considering all poses): Color code: OATP1B1 substituents….blue, OATP1B3 substituents….magenta, OATP2B1 substituents….green.

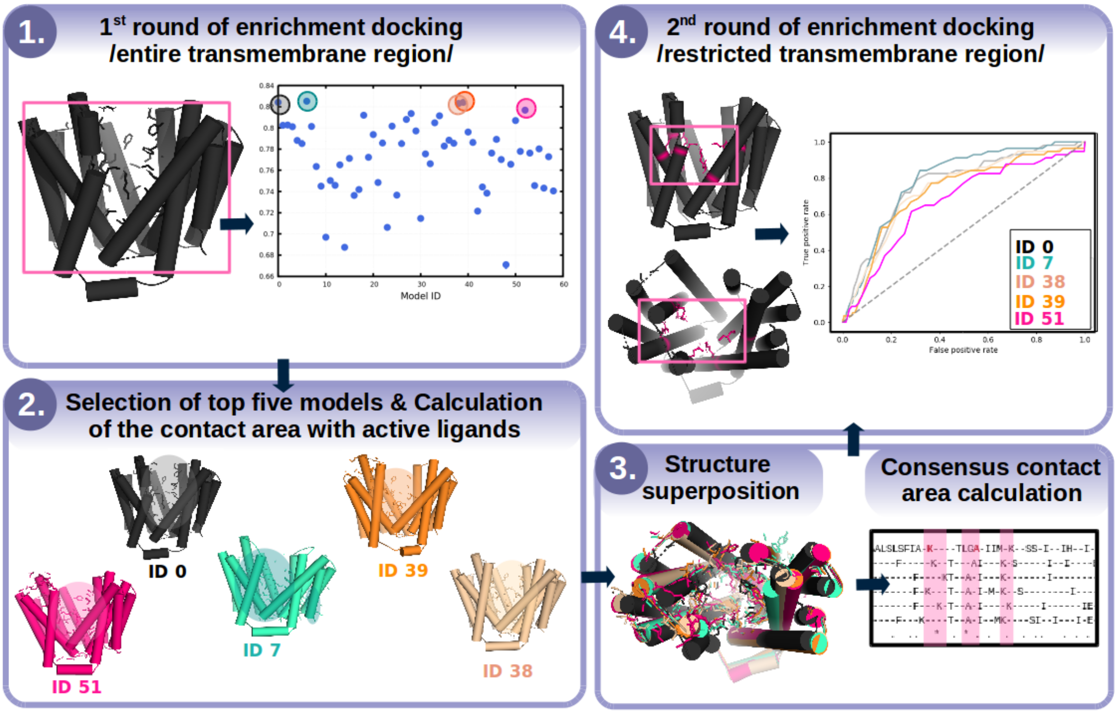

Figure S2. Schematic overview of the enrichment docking procedure.

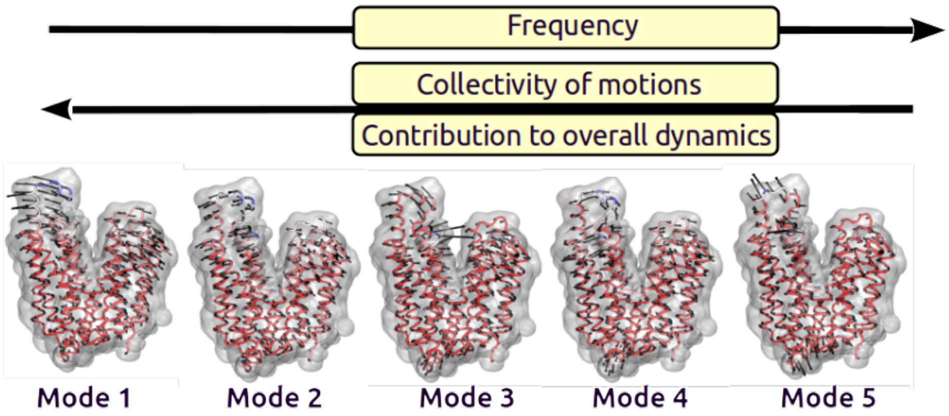

Figure S3. Five lowest frequency modes calculated for the selected template (Fucose transporter, PDB ID 3o7q): Black arrows in the structures indicate the magnitude and directionality of the fluctuation vectors.

a) b) c)

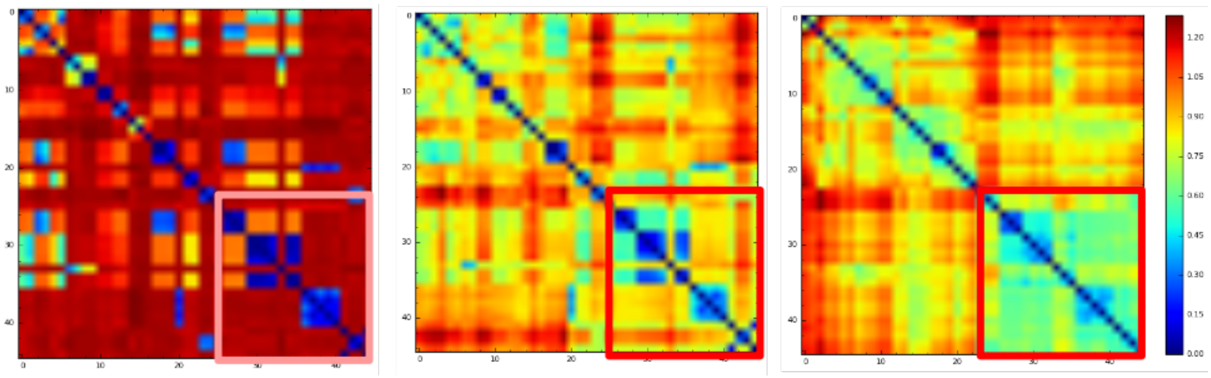

Figure S4. Covariance matrices showing a) sequence (Hamming distance), b) secondary structure (RMSD), and c) dynamic similarity between retrieved MFS transporters: The cluster of dynamically analogous transporters is shown by the red square in the matrices. Blue color indicates a strong similarity (identity), whereas red corresponds to complete dissimilarity. The sequence-based matrix (a) and secondary structure-based matrix (b) are reordered according to the dynamics-based matrix (c) to demonstrate that the sequentially and/or structurally divergent proteins might still share comparable intrinsic dynamics. PDB IDs of the structures unravelled by the similarity in their intrinsic dynamics are the following: 3o7q (reference structures), 4m64, 3wdo, 4gby, 4gbz, 4gc0, 4zwc, 4zw9, 5c65, 4jre, 4zwb, 4oaa, 4zyr, 4gxb, 3o7q, 5gxb.

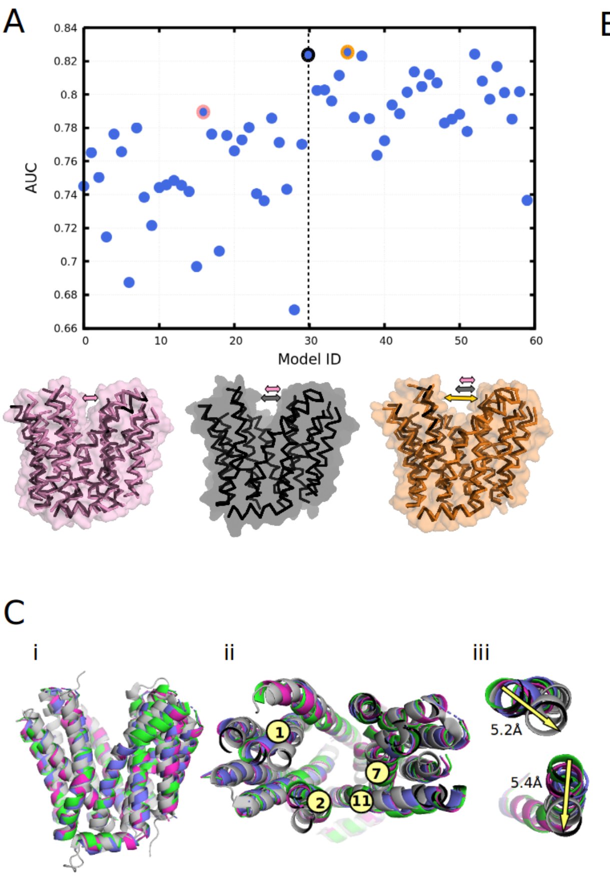

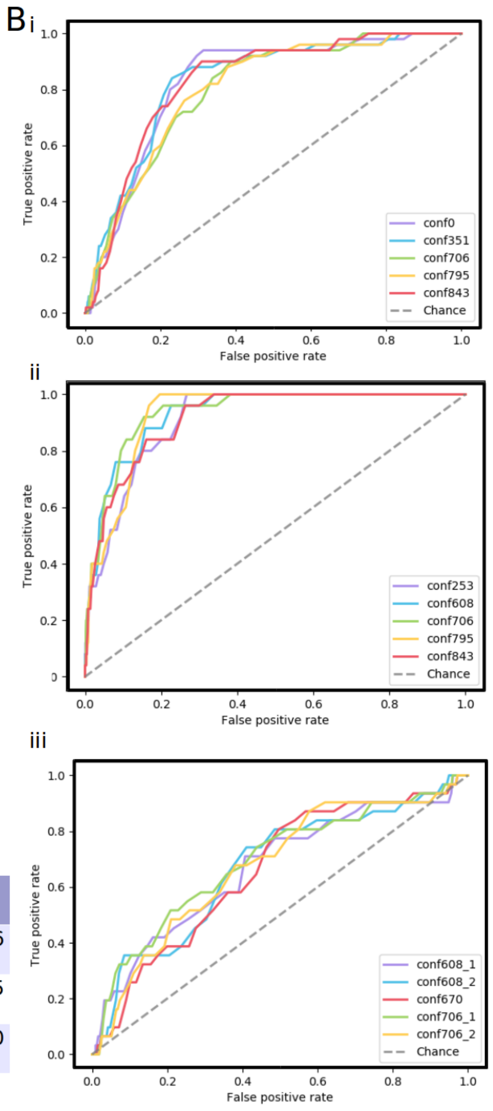

Model	Average RMSD	TMH1	TMH2	TMH7	TMH11
OATP1B1	2.8	4.5	5.5	2.9	3.6
OATP1B3	3.1	5.3	4.8	3.1	4.5
OATP2B1	3.2	5.8	5.9	3.4	4.0

Figure S5. Enrichment docking into OATP1B1/OATP1B3/OATP2B1 structural models: *(A)* Distribution of AUC values for OATP1B1 models from the first round of enrichment docking. The models are ordered by ascending value of their radius of gyration. The highlighted points in the plot correspond to the models depicted below the plot. The arrows indicate the degree of openness of the individual transporter structures. *(B)* ROC curves of the first docking run for the top five (i) OATP1B1, (ii) OATP1B3, and (iii) OATP2B1 structural models, respectively. *(C)* (i) Prioritized models for OATP1B1 (blue), OATP1B3 (magenta), and OATP2B1 (green) compared to the initial template (gray). Figure (ii) highlights TMHs with the highest variability. Figure (iii) depicts an out-of-plane motion of TMH1 and TMH2 compared to the initial template, as indicated by yellow arrows. The table lists distances [in Å] for TMH1 (C(55/76)---C(52) distance), TMH2 (C(66/87)---C(62) distance), TMH7 (C(361/396)---C(283) distance), and TMH11 (C(596/623)---C(407) distance) between the initial template and the final models.

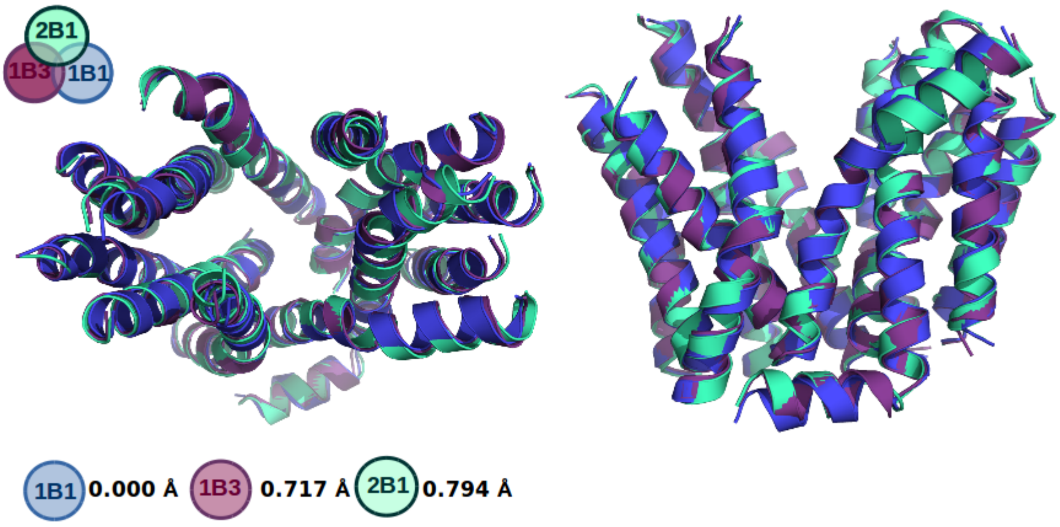

Figure S6. Comparison of the top prioritized structural models for OATP1B1 (the blue structure), OATP1B3 (the magenta structure), and OATP2B1 (the green structure): An average RMSD was calculated (OATP1B1 was defined as a reference structure for RMSD calculation).

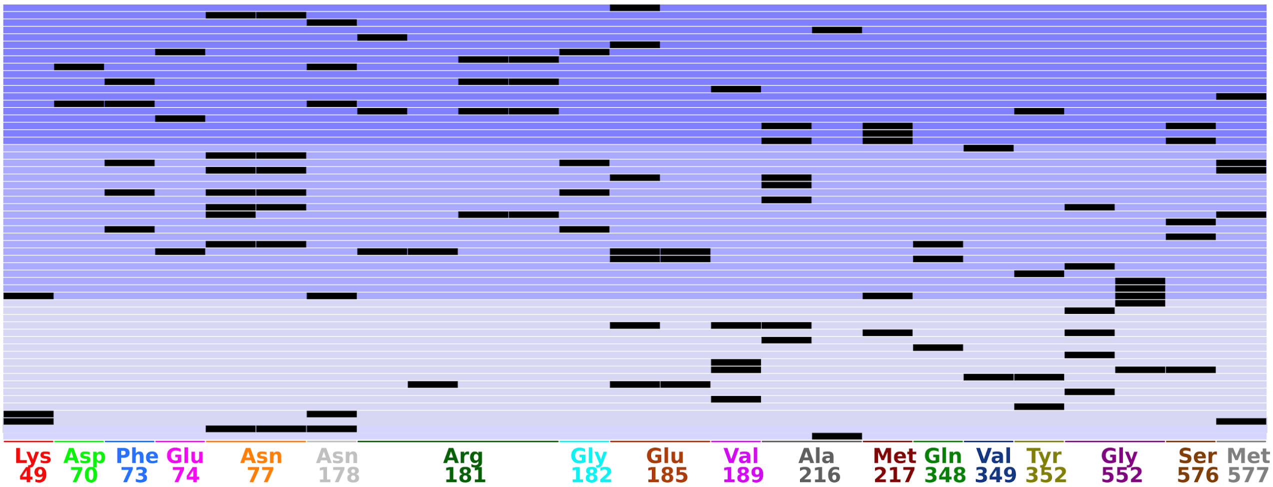

Figure S7. Protein-Ligand Interactions Fingerprints for top three enriched OATP1B1 clusters: Distinct clusters are highlighted in different colors.

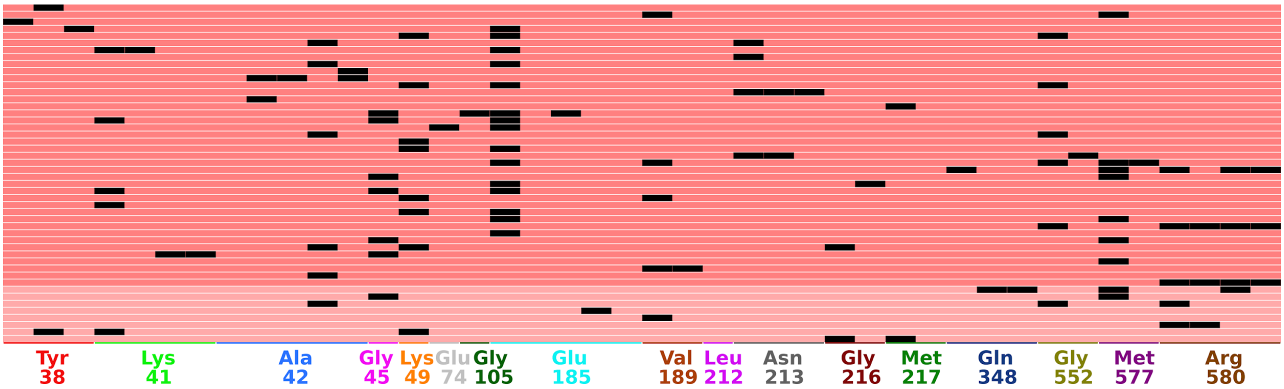

Figure S8. Protein-Ligand Interactions Fingerprints for top two enriched OATP1B3 clusters: Distinct clusters are highlighted in different colors.

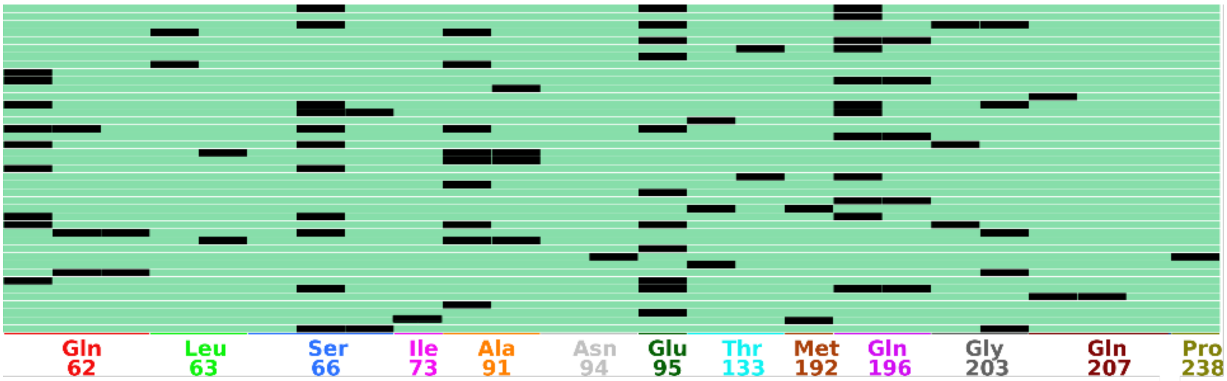

Figure S9. Protein-Ligand Interactions Fingerprints for the top enriched OATP2B1 cluster.

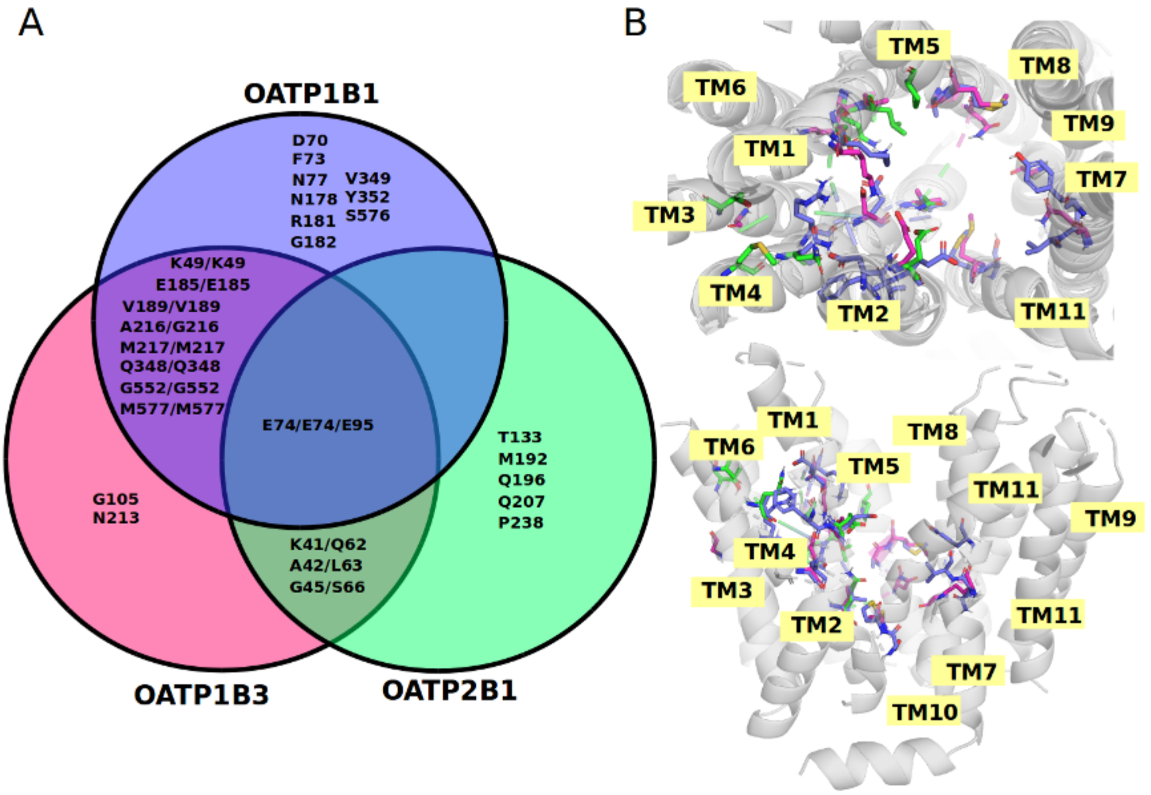

Figure S10. Key amino acid residues in OATP1B1/OATP1B3/OATP2B1 predicted to interact with steroid analogs: (A) Shared and distinct key protein ligand interactions. (B) Visualization of the key interacting residues within the transmembrane region (OATP1B1 residues shown in blue, OATP1B3 in magenta, OATP2B1 in green).

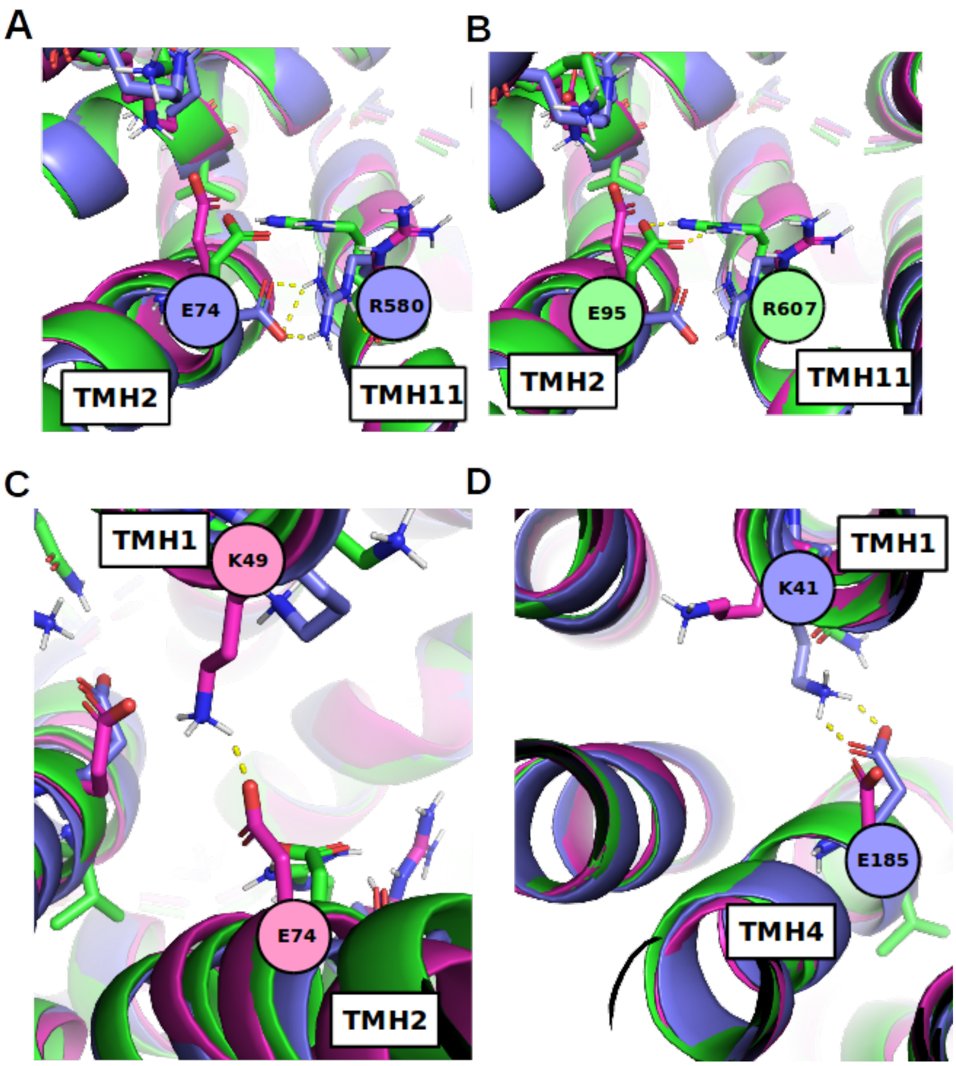

Figure S11. Identified intramolecular salt bridges in hepatic OATPs:

(A) GLU74(TMH2)-ARG580(TMH11) salt bridge in OATP1B1;

(B) GLU95(TMH2)-ARG607(TMH11) salt bridge in OATP2B1;

(C) LYS49(TMH1)-GLU74(TMH2) salt bridge in OATP1B3;

(D) LYS41(TMH1)-GLU185(TMH4) salt bridge in OATP1B1;

Color code: OATP1B1 = the blue structure, OATP1B3 = the magenta structure, OATP2B1 = the green structure

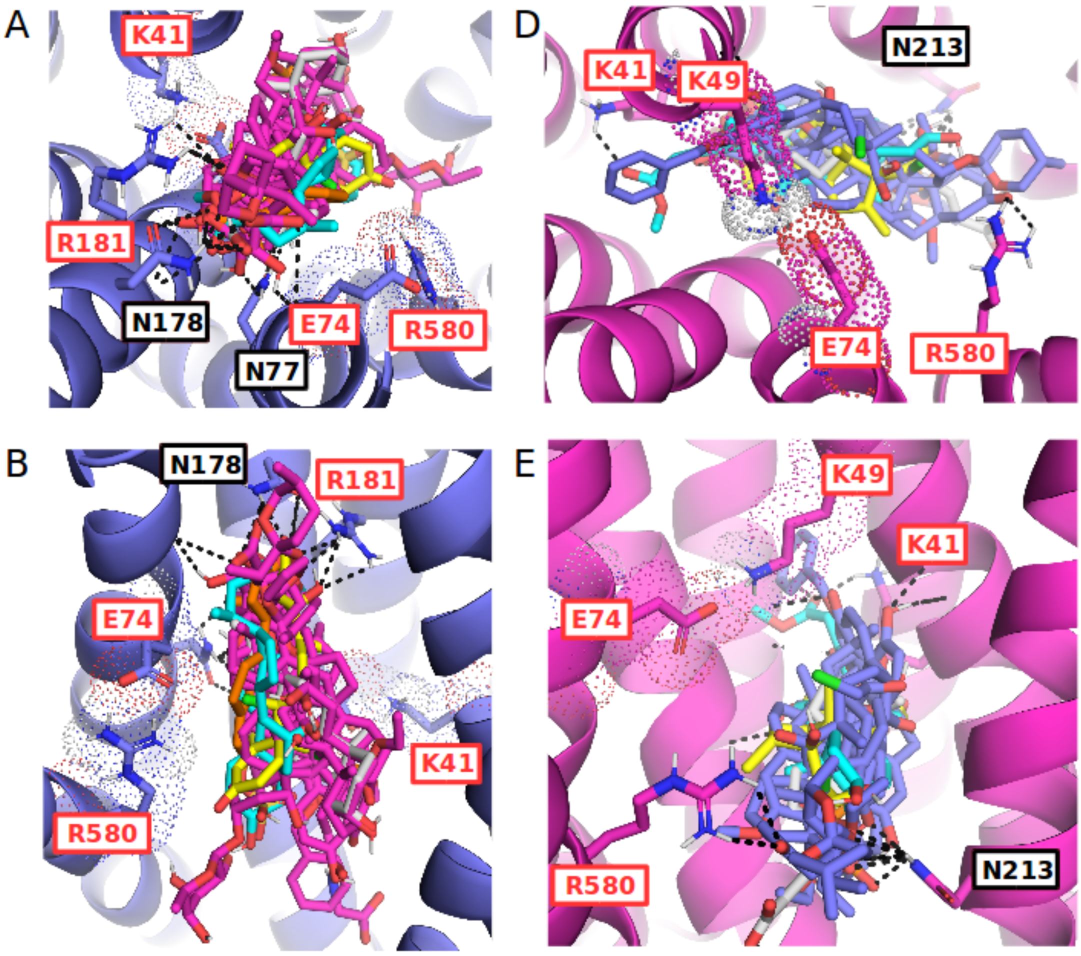

Figure S12. Comparison of interactions of steroids in the N-terminal binding site of OATP1B1 (blue structure) and OATP1B3 (magenta structure): Top ranked poses for all inhibitors belonging to the corresponding cluster are depicted in top view (A and D) and side view (B and E). C) shows the N-terminal binding pose of **E-3-S (**a selective OATP1B1 inhibitor**) in OATP1B1**; D) shows the N-terminal binding pose of **digoxin (**a selective OATP1B3 inhibitor**) in OATP1B3.** Hydrogen bonds are visualized via black dashed lines. Residues labeled in red were validated via mutagenesis studies published in the literature. Dotted surfaces around certain residues visualize van der Waals radii in order to highlight residues that are forming intramolecular salt bridges between protein residues (K41, K49, E74, E185, R580).

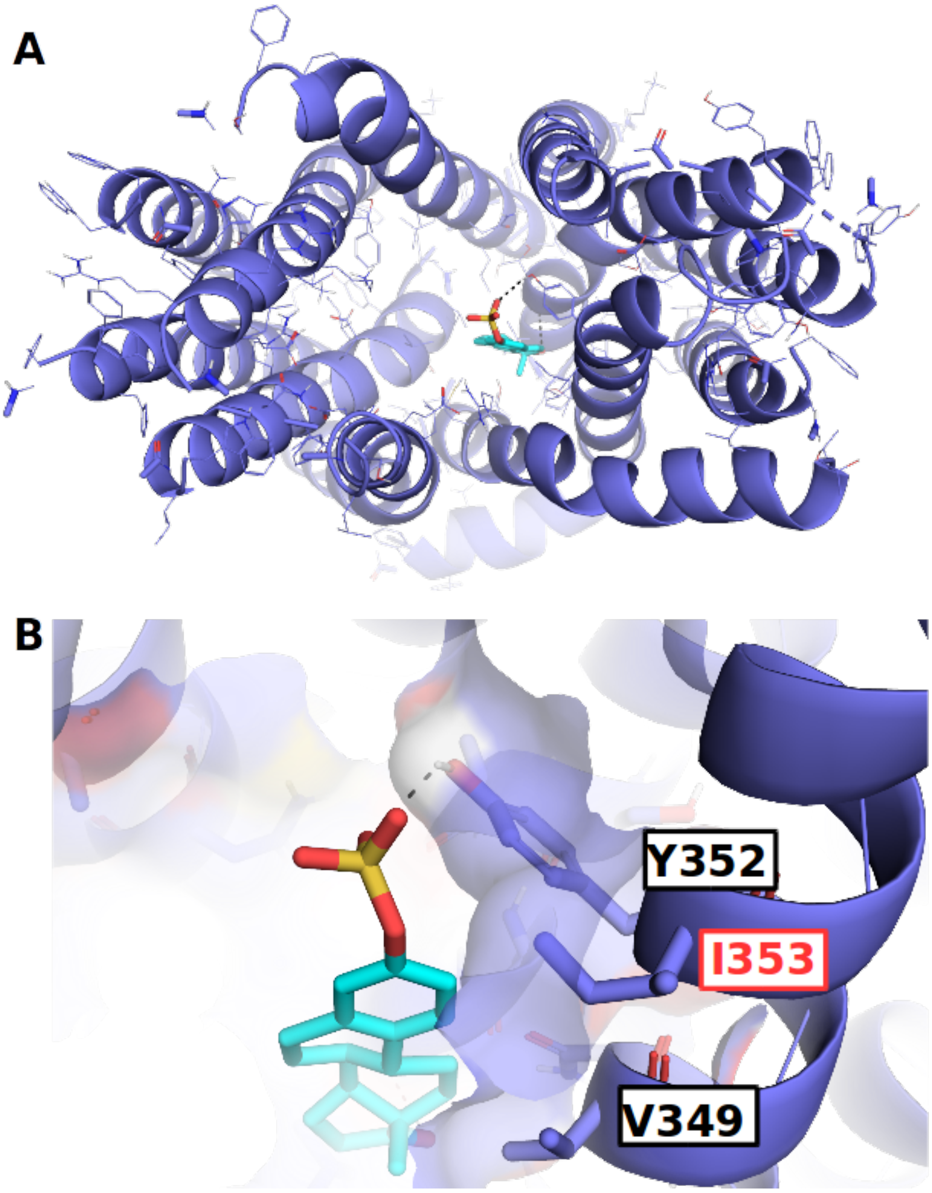

Figure S13. Binding mode of E-3-S in OATP1B1 suggesting OATP1B1 selectivity: (A) top view, (B) side view. Hydrogen bond is visualized by black dashed lines. The residue labeled in red is a known site of single nucleotide polymorphism.

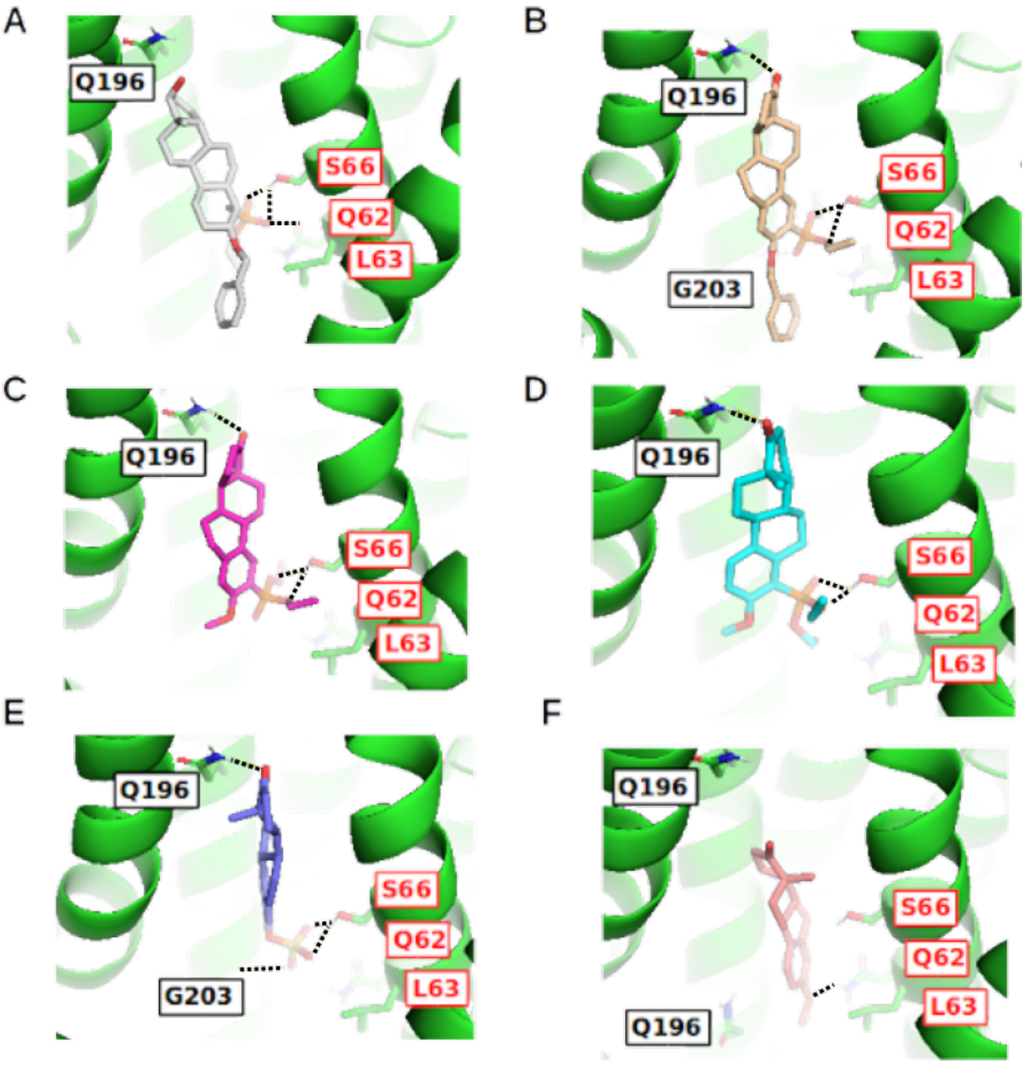

Figure S14. Binding modes of selected 13α-estrone derivatives in OATP2B1:
A) compound **12** (light gray, IC_{50}=0.75µM); **B)** compound **13** (light pink, IC_{50}=3.18µM); **C)** compound **15** (magenta, IC_{50}=2.96µM); **D)** compound **16** (cyan, IC_{50}=2.74µM); **E)** **E-3-S** (blue, IC_{50}=10.00µM); **F)** compound **2** (brown, IC_{50}=5.41µM). Hydrogen bonds and/or other polar contacts are visualized via black dashed lines. Residues labeled in red were validated via mutagenesis studies published in the literature.

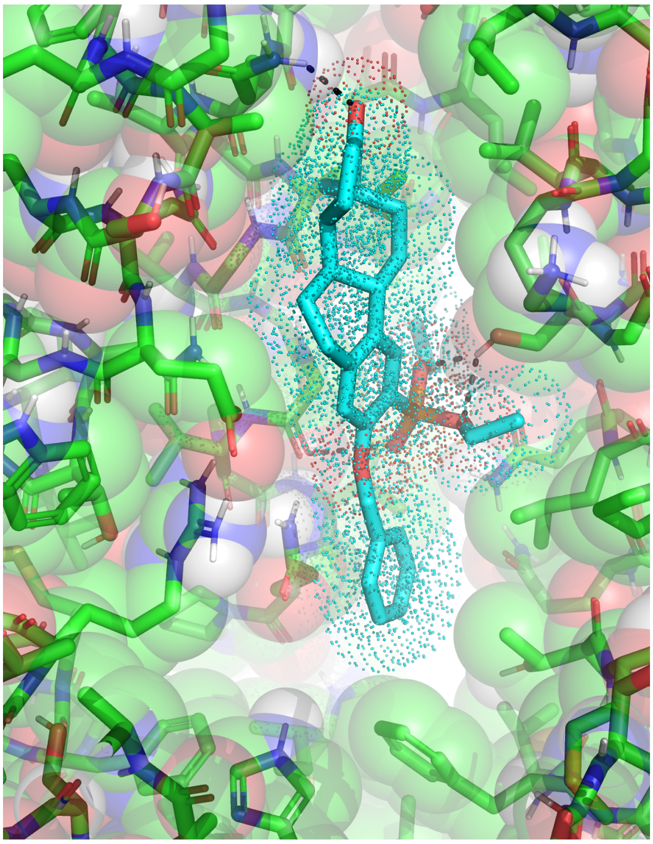

Figure S15. Docking pose of compound 11 visualized with van der Waals radii of the ligand and its surrounding environment: H-bonds with Ser66 and Gln196 are shown. The benzyloxy substituent is pointing towards a hydrophobic pocket. Color code: docked compounds = cyan (carbon), red (oxygen), orange (phosphore), OATP2B1 = green (carbon), red (oxygen), blue (nitrogen). Hydrogen bonds are indicated by black dashed lines.

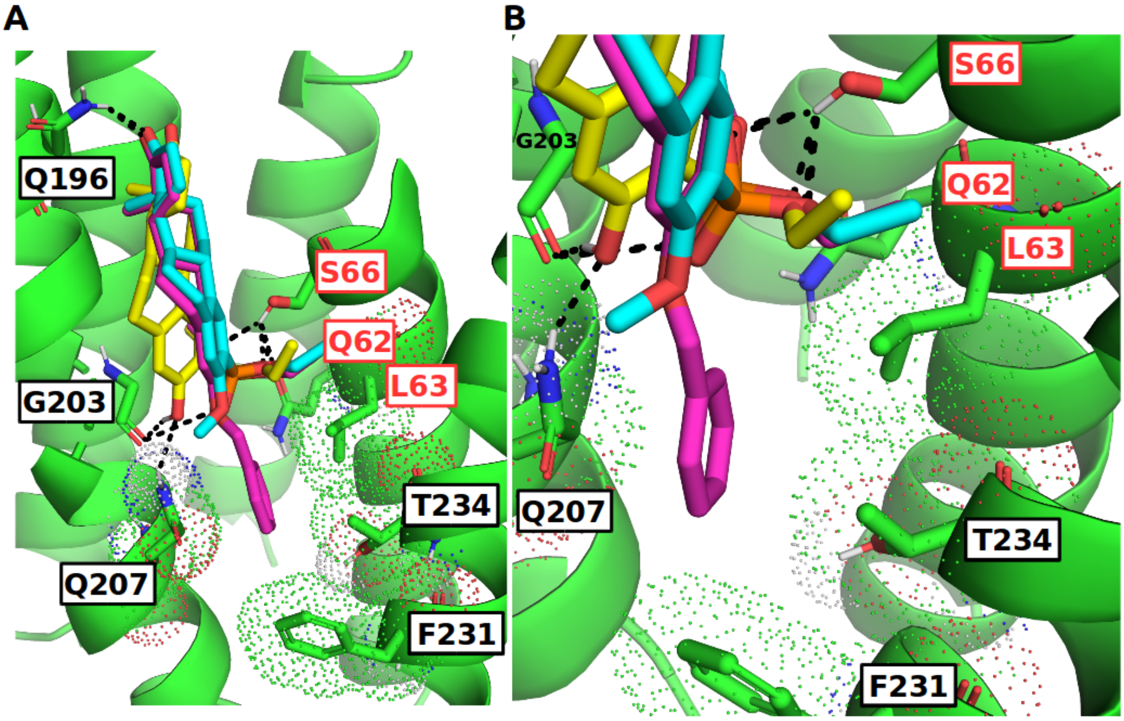

Figure S16. Binding modes of R-2 phosphonated 13α-estrone derivatives in OATP2B1: Compound **11** (magenta, IC_{50}=0.18), compound **13** (yellow, IC_{50}=3.18), and compound **15** (cyan, IC_{50}=2.96). Hydrogen bonds are visualized by black dashed lines. Residues labeled in red were validated via mutagenesis studies. Potential hydrophobic/surface stabilizing interactions of several residues are visualized via a dotted surface (LEU63, Q207, F231, and T234, respectively).

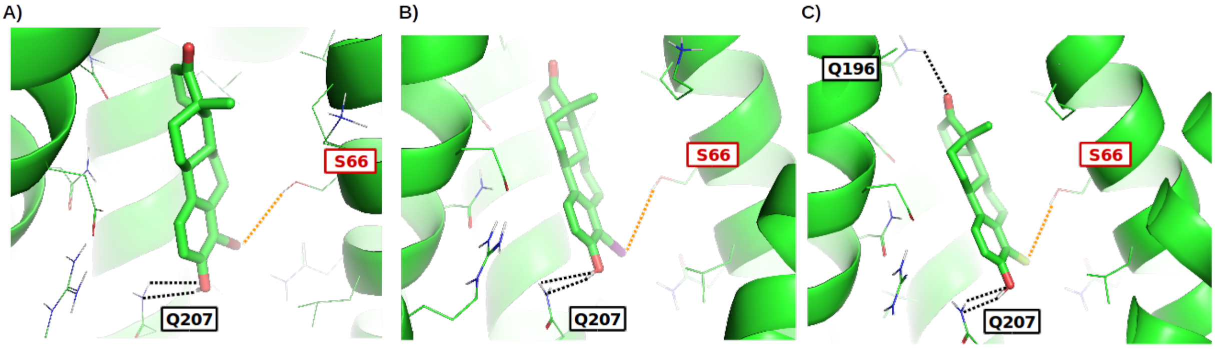

Figure S17. R-4 halogenated 13α-estrone derivatives binding in close proximity to SER66 in OATP2B1: The interaction angle C19-Cl1---OG(S66) is approximately 110°, thus the likelihood for halogen bond formation is decreased. A) Br-derivatives (compound **6)**, B) I-derivative (compound **8**), and C) Cl-derivative (compound **10**) are depicted. Color code: docked compounds = green (carbon), red (oxygen), limegreen (chloride), brown (bromide), purple (iodine). Residues labeled in red were validated via mutagenesis studies.

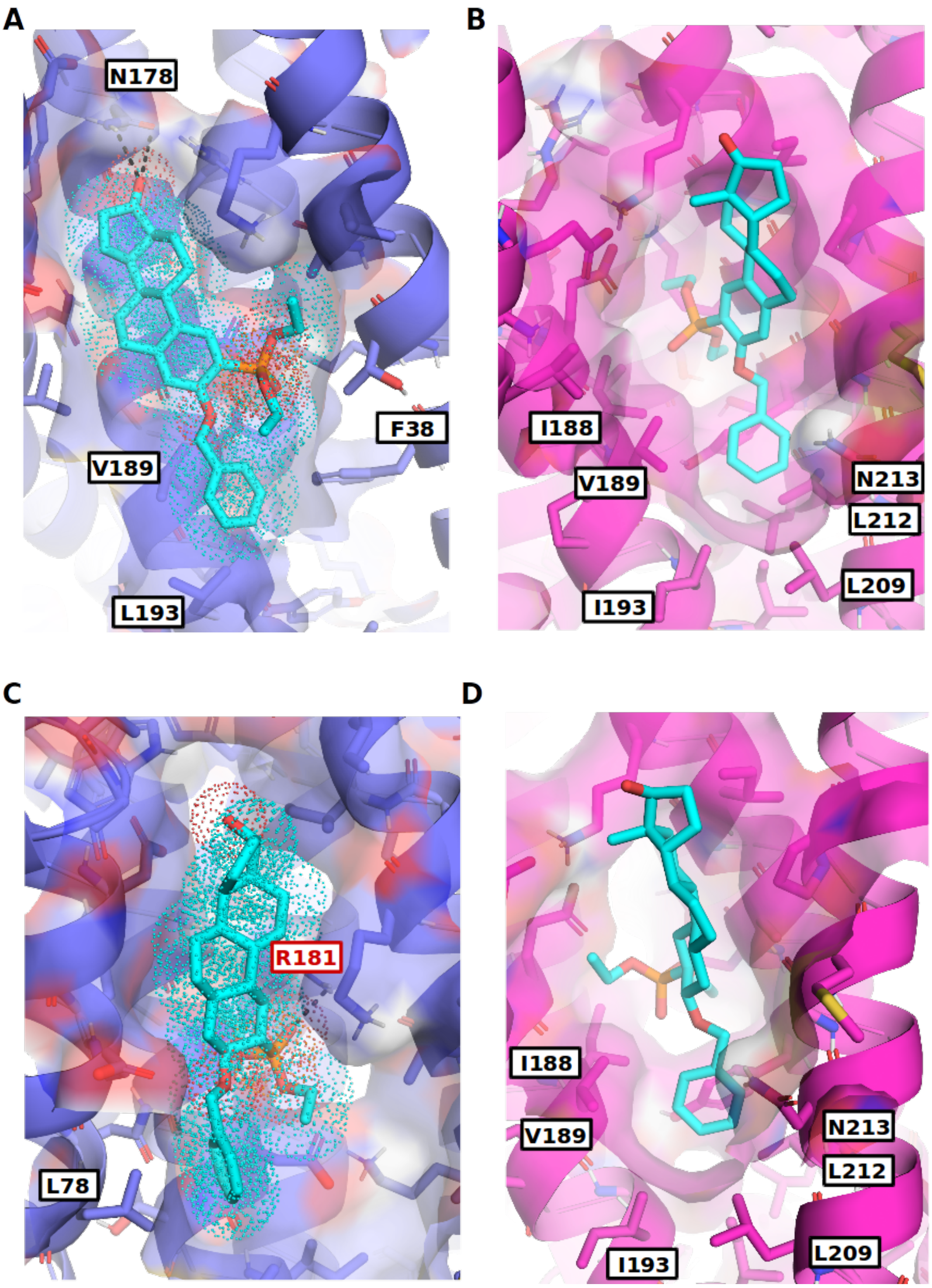

Figure S18. Binding of compound 11 (Figure A and B) and compound 12 (Figure C and D) in OATP1B1 (blue) and OATP1B3 (magenta) transporters.

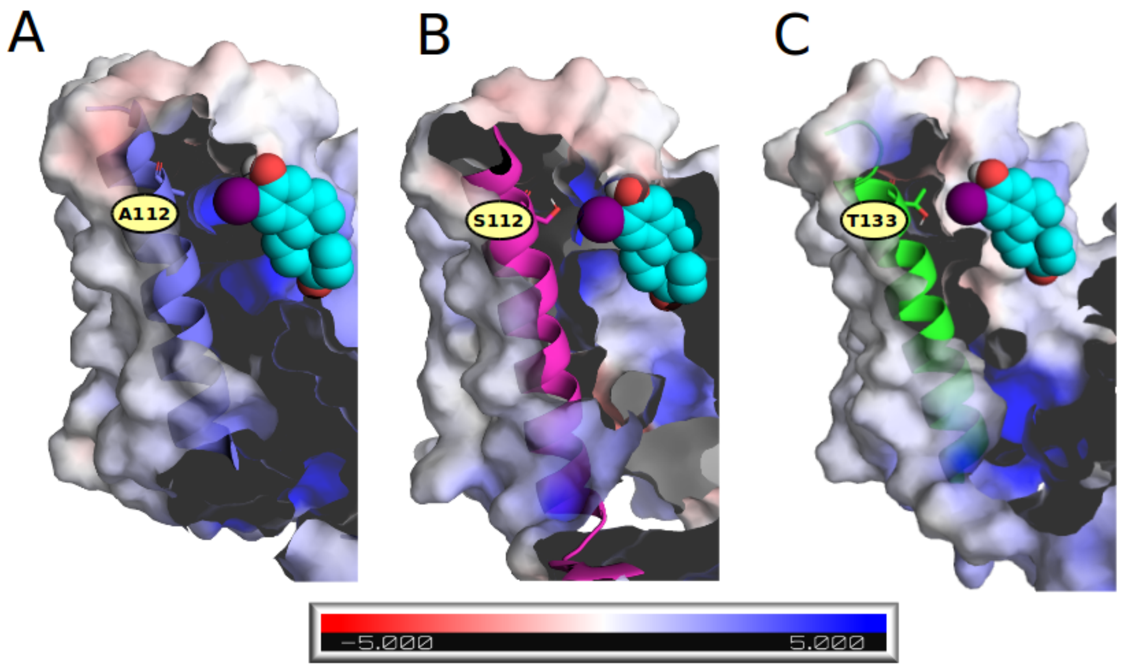

Figure S19. Putative halogen binding site in (A) OATP1B1 (B) OATP1B3 compared to the docked pose in (C) OATP2B1. Color code: docked compound = cyan (carbon), red (oxygen), purple (iodine), OATP1B1 = blue, OATP1B3=magenta, OATP2B1 = green.

Description of Supplementary Data Files

All Supplementary Files are also available from GitHub:
https://github.com/AlzbetaTuerkova/EnsembleDocking

File S1. Alignment file used for the comparative modeling of OATP1B1 (‘OATP1B1.ali’).

File S2. Alignment file used for the comparative modeling of OATP1B3 (‘OATP1B3.ali’)

File S3. Alignment file used for the comparative modeling of OATP2B1 (‘OATP2B1.ali’)

File S4. CSV file listing steroid analogs gathered from the open data sources:
For each measurement, the respective target (OATP1B1, OATP1B3, OATP2B1), InChIKey, bioactivity value per compound in μM, canonical smiles, and compound name are given.

File S5. Python script for retrieving the maximum common substructure from 3D ligand structures.

```
import glob
import sys
from rdkit import Chem
from rdkit.Chem import rdFMCS



# Find all .mol files in a current directory

mol_files = glob.glob('*.mol')
mol_list= []

# create a list of input molecules

for mol in mol_files:
      single = Chem.MolFromMolFile(mol)
      mol_list.append(single)

# find maximum common substructure (bond order is set to be flexible)

res = rdFMCS.FindMCS(mol_list,
bondCompare=rdFMCS.BondCompare.CompareAny).smartsString
pattern = Chem.MolFromSmarts(res)


# read a molecule in mol format

m = Chem.MolFromMolFile(sys.argv[1])
```

```
conf = m.GetConformer()
sub = m.GetSubstructMatch(pattern)

# get coordinates of a maximum common substructure of a molecule

print(len(sub))
print('')
for s in sub:
      coordinates=conf.GetAtomPosition(s)
      print(str(m.GetAtoms()[s].GetSymbol()) + " " + str(coordinates.x) + "
" + str(coordinates.y) + " " + str(coordinates.z))
```

File S6-S8. Structural models for OATP1B1, OATP1B3, and OATP2B1.
